# Role of Biological Control in Management of Invasive Exotic Arthropod Pests and Weeds in India

**DOI:** 10.3390/insects17010061

**Published:** 2026-01-01

**Authors:** Rangaswamy Muniappan, Kesavan Subaharan, Krishnan Selvaraj, Muthusamy Sampathkumar, Satya Nand Sushil

**Affiliations:** 1 Center for International Research, Education, and Development, Virginia Tech, Blacksburg, VA 24061, USA; 2 ICAR-National Bureau of Agricultural Insect Resources, Bellary Road, Bengaluru 560024, Indiaento_sam@yahoo.co.in (M.S.);

**Keywords:** biological control, history, successes, invasive insect pests, invasive weeds, India

## Abstract

Classical biological control of exotic invasive weeds first took place in India in 1795. Thus far, a total of 174 natural enemies have been imported into India, and out of these, 77 have established themselves in the field. Twelve exotic insect pests and four weeds were successfully controlled with a combination of classical, augmentative, and conservation biological control. Additionally, eight insect pests and one weed were substantially controlled. Augmentative biological control has been adopted as per the needs and availability of resources. Conservation biological control is ubiquitous and has been facilitated by the adoption of integrated pest management. In the past, biological control activities were sporadic; however, since 1977, the Indian Council of Agricultural Research—National Bureau for Agricultural Insect Resources has been regularly implementing classical biological control of invasive agricultural insect pests of economic importance. Unfortunately, the importance given to invasive weeds and insect pests of natural resources has fallen behind in recent years.

## Simple Summary

Invasive species can cause loss of biodiversity and agricultural productivity, adversely affecting human and environmental health. Biological control of exotic invasive insect pests and weeds has been practiced for more than two centuries in India. It has provided control in the categories of excellent (16), substantial (9), and partial (4) of exotic insect pests and weeds. Further, biological control in association with other components of integrated pest management has contributed to the decline in use of chemical pesticides in the past decade and a half in India, leading to sustainable development and environmental safety.

## 1. Introduction

Biological invasions pose a serious threat to biodiversity, agricultural productivity, human health, and environmental stability [1]. The socio-economic impact of biological invasions is staggering. India incurred losses between USD 127.3 billion and USD 182.6 billion from 1960 to 2020, averaging USD 2.1 to USD 3 billion annually. These costs stem from damage to agriculture, forestry, and ecosystems, as well as the expense of managing invasive species [2]. Invasive species often arrive without their natural enemies, giving them a competitive edge over native species. Despite quarantine regulations, their entry continues to rise due to globalization, climate change, migration, plant and animal trade, and other factors. Quarantine measures may delay invasions, but they rarely prevent them. The documentation of common prickly pear *Opuntia monocantha* (Wildenow) (Haworth) (Cactaceae) from northern India dates to the 1780s. Subsequently, erect prickly pear, *Opuntia stricta* (Haworth) and red-flower prickly pear, *Opuntia elatior* (Miller) established in southern India. Lantana (*Lantana camara* L.) (Verbenaceae) in 1809, Siam weed, *Chromolaena odorata* (L.) (King and Robinson) (Asteraceae) in the 1840s, and water fern, *Salvinia molesta* (D.S. Mitchell) (Salvinaceae) were introduced in 1955 [3].

Early records of invasive insect pests accidentally introduced were woolly apple aphid, *Eriosoma lanigerum* (Hausmann) (Hemiptera: Aphididae) in 1889, coffee green scale, *Coccus viridis* Green (Hemiptera: Coccidae) in the 1890s, potato tuber moth, *Phthorimaea opercullela* (Zeller) (Lepidoptera: Gelechiidae) in 1906, San Jose scale, *Comstockaspis* (=*Quadraspidiotus*) *perniciosa* (Comstock) (Hemiptera: Diaspididae) in 1910, diamondback moth, *Plutella xylostella* L. (Lepidoptera: Plutellidae) in 1914, and cottony cushion scale, *Icerya purchasi* Maskell (Hemiptera: Monophlebidae) in 1928. In a recent review, Sushil et al. [4] listed 36 invasive insect species in India. To highlight the increase in incidence of invasive species in the past 20 years (2006–2025), a list of 25 species is given in the Supplementary File (Table S1).

Biological control plays a major role in the management of invasive species, and all three strategies of biological control—classical, augmentative, and conservation—have been involved. Classical biological control is a favored frontline strategy for managing invasive species, particularly when their associated natural enemies are present in the native habitats, where these species typically pose no threat. Augmentative control has two strategies: inoculative and inundated release of natural enemies. Agents for augmentative biological control are selected based on their efficacy, biology, ease of rearing, cropping situation, seasonality, and cultural practices. It is adopted to suppress pests after they have established themselves by releasing laboratory-reared natural enemies. Both introduced and native natural enemies, based on their efficacy in tackling a target insect pest or weed, are utilized in this strategy. Conservation biological control involves preserving native, naturalized, and introduced natural enemies by selective use of insecticides, environmental modification by enhancing vegetational diversity, and habitat management [5,6]. While the management of a few species of invaded insect pests could be attributed to conservation biological control, it has most often been associated with classical and augmentative biological control. Here, we present a brief history of biological control action plans implemented in India over the past two centuries. We addressed invasive arthropod pests and weeds, natural enemies introduced, local natural enemies involved, augmentative and conservation biological control activities, notable successes, lessons learned, existing gaps in the system, and potential opportunities for future interventions.

## 2. Biological Control

### 2.1. History

Classical biological control of invasive weeds has a long history in India. In 1795, control of *O. monocantha*) with the introduction of the cochineal insect *Dactylopius ceylonicus* (Green) (Hemiptera: Dactylopiidae), which was originally introduced for production of cochineal dye, was serendipitously recognized. This was the first documented record of biological control of an invasive plant in the world [7]. Based on this achievement, *D. ceylonicus* was formally shipped from India to Sri Lanka for biological control of *O. monocantha* in 1865, resulting in the first documented intentional transfer of an agent for classical biological control [8]. In the early 1900s, recognizing the seriousness of invasive weeds, the Government of India deputed Mr. Ramachandra Rao from 15 November 1916 to 31 March 1919 to study the distribution and natural enemies of *L. camara* throughout India and Myanmar [9]. He reported 148 local herbivores, including two fortuitously introduced ones. Subsequently, the lantana pod fly, *Ophiomyia lantanae* (Froggatt) (Diptera: Agromyzidae) from Hawaii was introduced in 1921 [10]. In 1926, another cochineal insect *Dactylopius opuntiae* (Cockerell) (Hemiptera: Dactylopiidae), was introduced to India from Sri Lanka for control of *O. stricta* and *O. elatior* [11]. Afterwards, biological control of invasive weeds was carried out sporadically [12] by the forestry department, the Commonwealth Institute of Biological Control (CIBC) (now, the Center for Agriculture and Biosciences International (CABI)) station in India and institutions of the Indian Council of Agricultural Research (ICAR). However, relatively little work has been completed on new introductions of bioagents against weeds since the 1980s [13].

Classical biological control of invasive insect pests in India started when coffee green scale *Coccus viridis* Green (Hemiptera: Coccidae) was accidentally introduced to southern India in the 1890s. In 1898, Howard O. Newport, a planter and an amateur entomologist from Lower Pulneys, Madurai district in southern India, introduced *Cryptolaemus montrouzieri* Mulsant (Coleoptera: Coccinellidae) from Australia with the support of the United Planters’ Association of Southern India and the Government of Madras [14] to control *C. viridis*. In addition to the targeted pest, it has proven to be an effective agent for controlling mealybugs and soft scales [15,16]. In 1929, for control of cottony cushion scale *I. purchasi*, the Australian lady beetle *Novius* (*Rodolia*) *cardinalis* (Mulsant) (Coleoptera: Coccinellidae) was introduced via South Africa [17]. The parasitoid, *Aphelinus mali* (Haldimann) (Hymenoptera: Aphelinidae), was introduced from England in 1930 for control of woolly apple aphid, *E. lanigerum* [18]. Two parasitoids, *Bracon gelechiae* (Ashm.) (Hymenoptera: Braconidae) and *Trichogramma australicum* Gir. (Hymenoptera: Trichogrammatidae), were introduced in 1944 and 1948, respectively, for control of potato tuber moth, *P. operculella* [17]. From 1957 to 1987, the CIBC sub-station in Bengaluru carried out classical biological control research activities in India, and in 1977, the All India Coordinated Research Project on Biological Control of Crop Pests and Weeds was started, which eventually morphed into ICAR–National Bureau of Agricultural Insect Resources (NBAIR) to carry out biological control programs. As of 2022, there are 361 biological control laboratories in India, consisting of 35 Central Integrated Pest Management Centers (CIPMCs) and 38 State Biological Control Laboratories (SBCLs) of the Directorate of Plant Protection, Quarantine and Storage (DPPQS), 49 Indian Council of Agricultural Research laboratories, 98 state agricultural university laboratories, and 141 private laboratories [19].

### 2.2. Invasive Insect Pests

Exotic insects and weeds introduced to India are presented below in chronological order of their introduction, with a brief review of exotic natural enemies introduced for their control, and local natural enemies recruited.

Woolly apple aphid, *E. lanigerum*, native to China, was accidentally introduced to India in 1889. It infests members of the family Rosaceae, with apple being the preferred host. An exotic parasitoid, *Aphelinus mali* was introduced from England in 1930 [18]. Indian natural enemies of this aphid are *Coccinella septempunctata* L., *Coccinella transversoguttata* Faldermann, *Priscibrumus uropygialis* (Mulsant), *Harmonia dimidiata* (L.), *Chilocorus infernalis* Mulsant, *Adalia tetraspilota* (Hope), *Hippodamia variegata* (Goeze), *Oenopia conglobata* L., (Coleoptera: Coccinellidae), *Syrphus* spp. (Dipitera: Syrphidae), and *Chrysoperla zastrowi sillemi* (Esben-Petersons), *Chrysopa nigricornis* Burmeister (Neuroptera: Chrysopidae) [20,21,22]. The exotic parasitoid, *A. mali*, in association with native natural enemies, is providing substantial suppression of woolly apple aphid.

Coffee green scale, *C. viridis*, believed to be a native of Brazil, was introduced to southern India in the 1890s. It is a polyphagous pest affecting coffee, avocado, citrus, tea, mango, cassava, guava, and others. To control this pest, Mr. Howard O. Newport introduced *C. montrouzieri* in 1898 from Australia [14]. No follow-up work was taken up as Mr. Newport left India in 1899; however, in 1951, Puttarudriah et al. [23] found larvae and pupae of *C. montrouzieri* on the trunks of *Araucaria* pines around Bengaluru. Later, they also found *C. montrouzieri* specimens in the collections of the Mysore State Entomology Division that were taken from mealybugs in 1940 in Bengaluru. Indigenous parasitoids observed on *C. viridis* were *Coccophagus ceroplastae* (Howard), *C. cowperi* Girault, and *Coccophagus* sp. (Hymenoptera: Aphilinidae), *Anicetus annulatus* (Timberlake), *Encyrtus lecaniorum* (Mayr), *Metaphycus helvolus* (Compere), *M. lichtensiae* (Howard), and *M. maculatus* Agarwal (Hymenoptera: Encyrtidae) [24]. *Cryptolaemus montrouzieri*, in association with local natural enemies, has given excellent control of coffee green scale.

Potato tuber moth, *P. operculella*, a native of the Andes, South America, was introduced to Bangladesh in 1906 on potato seed, imported from Italy [25,26]. It is a major pest of potatoes in both the field and storage. A parasitoid *Bracon gelechiae* (Ashm.) (Hymenoptera: Braconidae) was imported from Canada in 1944. In 1948, *T. australicum* (Hymenoptera: Trichogrammatidae) (Origin: not known) was released in Nanjangud, Karnataka and Coonoor, Tamil Nadu [17]. In 1964 and 1965, five species of larval parasitoids, namely, *Agathis unicolor* (Schr.), *Apanteles scutellaris* Mues., *Orgilus lepidus* Muesebeck (Hymenoptera: Braconidae), *Campoplex haywardi* Blanch., *Temelucha* sp. (Hymenoptera: Ichneumonidae), and one egg-larval parasitoid, *Copidosoma uruguayensis* Tach. (Hymenoptera: Encyrtidae) from South America and the larval parasitoid *Apanteles subandinus* Blanchard (Hymenoptera: Braconidae) from North America, were introduced from the California (USA) station of CIBC [17]. Additional parasitoids *Orgilus parcus* (Turner) (Hymenoptera: Braconidae), and *Diadegma stellenboschense* Cameron from South Africa and *Diadegma raoi* Gupta (Hymenoptera: Ichneumonidae) from Cyprus were also introduced (year not known) [17]. In 1968, *Diadegma turcator* Aubert (Hymenoptera: Ichneumonidae) from Cyprus was introduced [27]. *Chelonus kellieae* Marsh (Hymenoptera: Braconidae) from Costa Rica in 1978, *Copidosoma koehleri* (Blanchard) (Hymenoptera: Braconidae) from Australia in 1976, and *Copidosoma desantis* (Annecke and Mynhardt) (Hymenoptera: Encyrtidae) from Peru were introduced [28]. The parasitoid, *Chelonus blackburni* (Cameron) (Hymenoptera: Braconidae), was introduced from the USA in 1976 by CIBC India [29]. A native parasitoid, *Trichogramma chilonis* (Ishii) (Hymenoptera: Trichogrammatidae) and the introduced parasitoid *C. blackburni* are used in augmentative releases for control of *P. operculella*. Substantial control of potato tuber moth has been achieved with the introduced and native parasitoids.

San Jose scale, *C. perniciosa*, is native to northern China and North Korea [15]. It appeared in India in 1910, possibly introduced along with the Japanese ornamental plant *Chaenomeles* (*Cydonia*) *japonica* (Thunb.) (Rosaceae) [30]. It is a pest of deciduous fruit trees. Exotic natural enemies introduced were *Aphytis diaspidis* Howard from the USA in 1960, *Aphytis* sp. nr. *diaspidis* How. (Hymenoptera: Aphelinidae) from Japan via USA in 1966, and *Encarsia perniciosi* (Tower) (Hymenoptera: Eulophidae), California strain from USA in 1958 and 1959, Chinese and Illinois (USA) strains via Switzerland in 1960, and Russian strain via France in 1960 [27]. Additionally, *Chilochorus kuwane* Silv. (Coleoptera: Coccinellidae) (Origin: Japan) and *Cybocephalus gibbulus* Erickson (Origin: Japan) (Coleoptera: Cybocephalidae) were imported from the CIBC station in Trinidad [17]. Of the two introduced parasitoids, only *E. perniciosi* was recovered from the field. However, *Aphytis* sp. *proclia* group was recorded from field surveys even though it was not intentionally introduced [31]. Local natural enemies, *Chilocorus circumdatus* Gyllenhal, *C. infernalis* Mulsant, *C. septempunctata*, *Sticholotis marginalis* Crotch (Coleoptera: Coccinellidae), *Aphytis lingnanensis* (Compere), *Aphytis paramaculicornis* (DeBach and Rosen) (Hymenoptera: Aphelinidae), and *Azotus kashmirensis* Narayanan (Hymenoptera: Azotidae) were reported on this pest from India [31]. Inoculative release of the parasitoids *E. perniciosi*, *A*. sp. *proclia* group, and the coccinellid *C. infernalis* at the beginning of the spring season has given excellent control of San Jose scale [15,31].

Diamondback moth, *P. xylostella*, a native of either Europe or South Africa, has established itself all over the world. The moth is a pest of cruciferous crops. It was first reported from India in 1914 [32]. The parasitoid, *Diadegma semiclausum* (Hellén) (Hymenoptera: Ichneumonidae) was introduced to India from Taiwan in 1994. Two egg parasitoids, *Trichogrammatoidea bactrae* (Nagaraja) from Taiwan [15] and *Trichogramma brassicae* Bezdenko (Hymenoptera: Trichogrammatidae) from Canada were introduced in 1992 and 2005, respectively [33]. Exotic parasitoids introduced for other pests, *Trichogramma evanescens* Westwood, *Trichogramma brasiliensis* (Ashmead), and *Trichogramma pretiosum* Riley (Hymenoptera: Trichogrammatidae) were reported to parasitize *P. xylostella* in India. *Cotesia vestalis* (Holiday) (Hymenoptera: Braconidae), *Oomyzus sokolowskii* Kurdjumov (Hymenoptera: Eulophidae), *Diadromus collaris* (Gravenhorst), and *D. semiclausum* (Hymenoptera: Ichneumonidae) are the key parasitoids of diamondback moth [27]. These introduced parasitoids have given substantial control of diamondback moth.

Lantana bug/Kew bug (*Insignorthezia insignis* (Browne)) (Hemiptera: Ortheziidae), a native of the neotropics introduced by accident to Nilgiris, southern India, from Sri Lanka in 1915 [9]. It is a pest of coffee, citrus and several other fruit and vegetable crops. It is also found to colonize weeds such as *L. camara*, *C. odorata*, *Mikania micrantha* Kunth (Asteraceae), and others. The coccinellid beetle, *C. montrouzieri*, has been reported to feed on this pest [15]. The release of *Hyperaspis pantherina* Fürsch (Coleoptera: Coccinellidae) in St. Helena, Hawaii, four African countries and Peru has resulted in substantial control of *I. insignis* [34,35].

Cottony cushion scale, *I. purchasi*, native to Australia, was introduced to India in 1928 via Sri Lanka [36]. It is a polyphagous pest of woody plants, notably the genus *Citrus*. The lady beetle, *N. cardinalis*, was introduced in 1930 (Origin: Australia) by receiving one consignment from South Africa, and a second one from California (USA) through the Commonwealth Institute of Entomology and a third one from Egypt. Shipments of the parasitoid *Cryptochetum iceryae* (Williston) (Diptera: Cryptochetidae) were received from California (Origin: Australia) in 1947 and 1948 and its field release did not result in establishment [17]. Local natural enemies found attacking *I. purchasi* were *Novius* (*Rodolia*) *fumidus* (Mulsant) (Coleoptera: Coccinellidae) [37], and *Stathmopoda melanochra* Meyr. (Lepidoptera: Stathmopodidae) [36]. Excellent control of *I. purchasi* was provided by *N. cardinalis* in association with local natural enemies.

Codling moth, *Laspeyresia* (*Cydia*) *pomonella* (L.) (Lepidoptera: Tortricidae), native to Europe and Northwestern Asia, was introduced to the Ladakh area of India in 1972 from the northwestern border of Pakistan and Afghanistan [38,39]. It is a serious pest of apple, pear, apricot, and walnut and is still confined to the union territory Ladakh. The egg parasitoid *Trichogramma embyrophagum* (Hartig) (Hymenoptera: Trichogrammatidae) was introduced from Germany and France in 1993 and 2007, respectively [33]. Egg parasitoids introduced for other pests including *T. evanescens*, *T. pretiosum* [40], and *Trichogramma cacoeciae* Marchal (Hymenoptera: Trichogrammatidae) [39] have also been reported to parasitize this pest.

Brown peach aphid, *Pterochloroides persicae* (Cholodkovsky) (Hemiptera: Aphididae) a native of China, established in the state of Punjab and the union territory Kashmir in 1970 [41]. Local natural enemies recorded were *Harmonia eucharis* (Mulsant), *C. septempunctata*, *H. variegata*, *Oenopia conglobata* (L.), *Priscibrumus uropygialis* (Mulsant), and *A. tetraspilota* (Coleoptera: Coccinellidae), and *C. zastrowi sillemi* (Neuroptera: Chrysopidae) [42].

Citrus mealybug, *Planococcus citri* (Risso) (Hemiptera: Pseudoccocidae) is Asiatic in origin but pantropical in distribution and found in some sub-tropical regions. It was reported from India in 1987 [43]. A parasitoid, *Leptomastrix dactylopii* Howard (Hymenoptera: Encyrtidae) of Brazilian origin was introduced to India from Trinidad in 1983 [15]. An indigenous parasitoid *Coccidoxenoides peregrinus* (Timberlake) (Hymenoptera: Encyrtidae), along with the introduced coccinellid *C. montrouzieri* were found attacking this mealybug. These three natural enemies have given excellent control of *P. citri*.

Leucaena psyllid, *Heteropsylla cubana* (Crawford) (Hemiptera: Psyllidae), is a native of Central America introduced to India from Sri Lanka in 1988 [44]. It is a pest of *Leucaena leucocephala* (Lam.) de Wit (Fabaceae); considered by some to be an invasive plant and others a beneficial fodder plant. Soon after finding *H. cubana* in India, *Curinus coeruleus* (Mulsant) (Coleoptera: Coccinellidae) (Origin: Mexico) was introduced from Thailand in 1988 [45]. Native predators, *Cheilomenes sexmaculata* (Fabricius) (Coleoptera: Coccinellidae) and *Pantala flavescens* (Fabricius) (Odonata: Libellulidae), were also reported to feed on this psyllid [46]. *Curinus coeruleus* alone has given excellent control of *H. cubana*.

American serpentine leafminer, *Liriomyza trifolii* (Burgess) (Diptera: Agromyzidae), a native of the eastern part of the USA was introduced to India in 1990 [47]. The parasitoid, *Diglyphus begini* (Ashmead) (Hymenoptera: Eulophidae) was introduced to India from California but it did not establish [48]. Local parasitoids *Hemiptarsenus varicornis* (Girault) and *Chrysonotomyia rexia* (Narendran, Galande and Mote) (Hymenoptera: Eulophidae) have been reported on *L. trifolii* in India [49]. This pest is under control by the indigenous natural enemies.

Coffee berry borer, *Hypothenemus hampei* (Ferrari) (Coleoptera: Curculionidae) is a native of West Africa introduced to India from Sri Lanka in 1990 [50]. Two parasitoids, *Cephalonomia stephanoderis* Betrem and *Prorops nasuta* Waterston (Hymenoptera: Bethylidae) were imported from Mexico in 1995. *Phymastichus coffea* LaSalle (Hymenoptera: Eulopidae) and *P. nasuta* were imported from Colombia in 1999 [51]. Efficacy of these introduced parasitoids is yet to be evaluated.

Spiralling whitefly, *Aleurodicus dispersus* Russell (Hemiptera: Aleyrodidae), a native of Central America and the Caribbean region was reported from India in 1994 [52]. Fortuitously introduced parasitoids reported on it are *Encarsia guadeloupae* Viggiani (Hymenoptera: Aphelinidae), *Encarsia* (?) *haitiensis* (Dozier), and *Encarsia dispersa* Polaszek (Hymenoptera: Aphelinidae). Viraktamath [48] and Mani and Krishnamoorthy [53] reported native predators *Axinoscymnus puttarudriahi* Kapur & Munshi, *C. sexmaculata*, *Chilocorus nigrita* (Fabricius), *C. montrouzieri*, *Pseudoaspidimerus trinotatus* (Thunberg), *Serangium parcesetosum* Sicard, *Scymnus nubilus* Mulsant (Coleoptera: Coccinellidae), *Cybocephalus* sp. (Coleoptera: Cybocephalidae), *Acletoxenus indicus* (Malloch) (Diptera: Drosophilidae), *Apertochrysa* *astur* (Banks), *Mallada boninensis* (Okamoto), *C. zastrowi sillemi* (Neuroptera: Chrysopidae), and *Notiobiella* sp. (Neuroptera: Hemerobiidae). Spiralling whitefly has been successfully suppressed by the exotic and native natural enemies.

Eucalyptus gall wasp, *Leptocybe invasa* (Fisher and La Salle) (Hymenoptera: Eulophidae), is a native of Australia that invaded Villuppuram district of Tamil Nadu, India in 2001 [54]. The parasitoid *Quadrastichus mendeli* Kim and La Salle (Hymenoptera: Eulophidae) was imported from Israel in 2008 and field released in 2010 in various parts of India. Also, native parasitoids *Megastigmus viggianii* (Narendran and Sureshan), *Megastigmus dharwadicus* (Narendran, Girish Kumar, and Vastrad) (Hymenoptera: Torymidae), and *Aprostocetus gala* (Walker) (Hymenoptera: Eulophidae) were recorded from Karnataka [54,55,56,57]. Introduced and native parasitoids have effectively suppressed *L. invasa*.

Sugarcane woolly aphid, *Ceratovacuna lanigera* (Zehntner) (Hemiptera: Aphididae), a native of Southeast Asia, established in West Bengal in 1958 and later in northeast India [58]. It invaded western India starting with Maharashtra in 2002, and subsequently reached southern India [59]. In 2004, *Encarsia flavoscutellum* Zehntner (Hymenoptera: Aphelinidae) was collected from Assam and released in Karnataka and other south Indian states. Local predators *Dipha aphidivora* (Meyrick) (Lepidoptera: Pyralidae) and *Micromus igorotus* (Banks) (Neuroptera: Hemerobiidae) and *Eupeodes confrater* Weidemann (Diptera: Syrphidae) were also recorded [60]. This aphid has been well managed by utilizing native natural enemies.

Erythrina gall wasp, *Quadrastichus erythrinae* (Kim) (Hymenoptera: Eulophidae) is a native of Africa. Tanzania is the putative source of the population that invaded Asia and the Pacific. It invaded Kerala, India in 2005 causing galls on leaves and tender stems of *Erythrina stricta* Roxb, (Fabaceae) [61]. In India, a species of *Aprostocetus*, previously misidentified as *Aprostocetus gala* Walker (Hymenoptera: Eulophidae), has been recorded as a potential parasitoid of the erythrina gall wasp. The parasitoid, *Eurytoma erythrinae* (Gates and Delvare) (Hymenoptera: Eurytomidae) was introduced to Hawaii, where it has provided effective control of the erythrina gall wasp [62]. Considering its host specificity and proven effectiveness, the possibility of introducing it to India may be considered if the damage by erythrina gall wasp persists.

Cotton mealybug, *Phenacoccus solenopsis* (Tinsley) (Hemiptera: Pseudococcidae) is a native of USA and it reached Punjab, India in 2006 [63] via Pakistan. It is a serious pest of cotton and has been reported on over 200 host plants. A parasitoid *Aenasius arizonensis* (Girault) (Hymenoptera: Encyrtidae) from the native region of this mealybug was fortuitously introduced to India in 2009 which provided effective control of this pest [64]. Local natural enemies recorded on this mealybug are *Hyperaspis maindroni* (Sicard), *Brumoides suturalis* (Fabricius), *C. sexmaculata*, *C. septempunctata*, *H. variegata*, *Nephus regularis* (Sicard, *Scymnus coccivora* (Ayyar), *C. montrouzieri* (Coleoptera: Coccinellidae), *C. zastrowi sillemi*, *Mallada* sp., (Neuroptera: Chrysopidae), *Encyrtus aurantii* (Geoffroy), *Anagyrus mirzai* (Agarwal and Alam), *Anagyrus kamali* Moursi, *Anagyrus dactylopii* (Howard), *Homalotylus albiclavatus* (Agarwal), *Metaphycus* sp., (Hymenoptera: Encyrtidae), *Aprostocetus bangaloricus* Narendran (Hymenoptera: Eulophidae), *Chartocerus kerrichi* (Agarwal) (Hymenoptera: Signiphoridae), *Pachyneuron leucopiscida* (Mani) (Hymenoptera: Pteromalidae) [64,65]. *Aenasius arizonensis* alone has successfully suppressed cotton mealybug infestations.

Papaya mealybug, *Paracoccus marginatus* (Williams and Granara de Willink) (Hemiptera: Pseudococcidae), native to Mexico, was accidentally introduced to India in 2008 [66]. It is a pest of papaya, mulberry, cassava, and several other crops. Three parasitoids from Puerto Rico of Mexican origin, *Acerophagus papayae* (Noyes and Schauff), *Anagyrus loeckei* (Noyes and Menezes), and *Pseudleptmastix mexicana* (Noyes and Schauff) (Hymenoptera: Encyrtidae), were introduced in 2010, of which *A. papayae* proved to be effective in suppressing this pest. Local predators *Spalgis epius* (Westwood) (Lepidoptera: Lycaenidae), *C. montrouzieri*, *Scymnus coccivora* Ramakrishna (Ayyar), *Nephus regularis* (Sicard), and *Slipinskiscymnus saciformis* (Motschulsky) (Coleoptera: Coccinellidae), *Gitonides* sp. (Diptera: Drosophilidae), and chrysopid species (Neuroptera: Chrysopidae) were reported on this pest [67]. *Acerophagus papayae* has successfully suppressed papaya mealybug.

Jack Beardsley mealybug, *Pseudococcus jackbeardsleyi* (Gimpel and Miller) (Hemiptera: Pseudococcidae) is a native of the neotropics recorded in Tamil Nadu, India in 2012 [68]. It is a polyphagous pest of fruit, vegetables, and ornamental crops. *Cryptolaemus montrouzieri*, *S. coccivora*, *N. regularis*, *S. saciformis* and *Scymnus spicatus* (Coleoptera: Coccinellidae), *Mallada boninensis* (Okamoto) (Neuroptera: Chrysopidae) and *S. epius* (Lepidoptera: Lycaenidae) have been reported to provide substantial control of this mealybug negating the need for classical biological control [69].

Madeira mealybug, *Phenacoccus madeirensis* (Green) (Hemiptera: Pseudococcidae) is a native of neotropics introduced to India in 2012 [70]. It is a polyphagous pest attacking many ornamental and fruit crops and weeds. Native and naturalized natural enemies recorded were *Nephus regularis* (Sicard) [71], *C. montrouzieri*, *C. sexmaculata*, *Scymnus* sp., *Jauravia* sp., *Novius* (*Rodalia*) sp., *Chilocorus* sp. *Nephus regualris* Sicard (Coleoptera: Coccinellidae), *S. epius* (Lepidoptera: Lycaenidae), *C. zastrowi sillemi* (Neuroptera: Chrysopidae), *Cacoxenus* (*Gitonides*) *perspicax* (Knab) (Diptera: Drosophilidae), *Diadiplosis* sp. (Diptera: Cecidomyiidae) [72], *Allotropa* sp., (Hymenoptera: Platygastridae), *Anagyrus amnestos* (Rameshkumar, Noyes, Poorani and Chong), *Anagyrus qadrii* (Hayat, Alam and Agarwal), *A. loecki* and *Anagyrus* sp. nov. nr. *sinope* (Noyes and Menezes) (Hymenoptera: Encyrtidae) [69]. A combination of both native and naturalized natural enemies are providing substantial control of Madeira mealybug.

Banana skipper, *Erionota torus* Evans (Lepidoptera: Hesperiidae) is a native of Southeast Asia. It invaded southern India in 2012 [73]. Egg parasitoids *Ooencytus pallidipes* (Ashmead) (Hymenoptera: Encyrtidae), *Agiommatus* sp. (Hymenoptera: Pteromalidae), *Anastatus* sp. (Hymenoptera: Eupelmidae), and *Telenomus* sp. (Hymenoptera: Scelionidae); larval parasitoids *Elasmus brevicornis* Gahan (Hymenoptera: Eulophidae), *Pediobius* sp. nr. *elasmi* (Ashmead) (Hymenoptera: Eulophidae), *Tetrastichus* sp. (Hymenoptera: Eulophidae), *Acropimpla* sp. nr. *nigroscutis* (Cameron) (Hymenoptera: Ichneumonidae), *Cotesia* sp. (Hymenoptera: Braconidae); and the pupal parasitoids *Senometopia* sp., *Winthemia sumatrana* (Townsend), and *Drino* (*Palexorista*) sp., (Diptera: Tachinidae), *Megaselia scalaris* (Loew) (Diptera: Phoridae); *Brachymeria lasus* (Walker) (Hymenoptera: Chalcididae) and an unidentified species of Tachinidae were reported from southern India [74,75]. It is another example of local natural enemies managing an invasive pest.

South American tomato leafminer, *Phthorimaea* (=*Tuta*) *absoluta* (Meyrick) (Lepidoptera: Gelechiidae) is a native of South America that reached Pune, India in 2014 [76]. Native and naturalized natural enemies of this leafminer recorded were *Nesidiocoris tenuis* (Reuter) (Hemiptera: Miridae), *Trichogramma achaeae* (Nagaraja and Nagarkatti), *T. pretiosum* and *T. bactrae* (Hymenoptera: Trichogrammatidae), *Neochrysocharis formosa* (Westwood) (Hymenoptera: Eulophidae), *Habrobracon* sp. (Hymenoptera: Braconidae) and *Goniozus* sp. (Hymenoptera: Bethylidae) [77].

Solanum whitefly, *Aleurotrachelus* (=*Aleurothrixus*) *trachoides* (Back) (Hemiptera: Aleyrodidae) is of neotropical origin and a pest of Solanaceae, Verbenaceae, and Convolvulaceae plants, and it was introduced to India in 2014 [78]. Accidentally introduced parasitoid *Encarsia cubensis* (Gahan) (Hymenoptera: Aphelinidae) has been recorded on this whitefly [79] and it is providing substantial control.

Tobacco thrips, *Thrips parvispinus* (Karny) (Thysanoptera: Thripidae), a native of southeast Asia, reached India in 2015 [80]. It is polyphagous but causes serious damage to pepper and other crops. A natural enemy *Menochilus sexmaculatus* (Fabricius) (Coleoptera: Coccinellidae) has been recorded to feed on this pest [81].

Rugose spiralling whitefly, *A. rugioperculatus* is a native of Central America that invaded southern India in 2016 [82]. The accidentally introduced parasitoids *E. dispersa* and *Encarsia guadeloupae* Viggiani (Hymenoptera: Aphelinidae) were found parasitizing this pest. Local predators observed on this pest were *A. astur* (Neuroptera: Chrysopidae), *Cybocephalus* sp. (Coleoptera: Cybocephalidae), *Diadiplosis* sp. (Diptera: Cecidomyiidae), *Jauravia pallidula* Motschulsky, *C. sexmaculata*, and *Stethorus* sp. (Coleoptera: Coccinellidae) [83,84]. This whitefly has been successfully controlled by the exotic and local natural enemies.

Bondar’s nesting whitefly, *Paraleyrodes bondari* Peracchi (Hemiptera: Aleyrodidae), of neotropical origin, was reported from India in 2018 [85]. No parasitoids were reported on this whitefly in India [86]. Predators *Cybocephalus indicus* (Tian and Ramani), *Cybocephalus nipponicus* Endrödy-Younga (Coleoptera: Cybocephalidae) and *Delphastus catalinae* (Horn) (Coleoptera: Coccinellidae) were reported feeding on it [87].

Fall armyworm, *Spodoptera frugiperda* (Smith) (Lepidoptera: Noctuidae), native to the Americas, was introduced to India in 2018 via Africa [88]. Even though it is a polyphagous pest, the population that reached India preferred maize the most. A parasitoid *Telenomus remus* Nixon (Hymenoptera: Scelionidae) introduced from Papua New Guinea to India in 1963 [16] for control of *Spodoptera litura* (Fabricius) (Lepidoptera: Noctuidae) and *Achaea janata* (L.) (Lepidoptera: Erebidae) has become a major natural enemy of *S. fugiperda*, supporting the theory of ‘New Association’ proposed by Hokkanen and Pimentel [89]. In addition, native and naturalized parasitoids include *T. chilonis* (Hymenoptera: Trichogrammatidae), *C. blackburni*, *Chelonus formosanus* (Sonan) (Hymenoptera: Braconidae), *Odentepyris* sp. (Hymenoptera: Bethylidae), *Aleiodes* sp., *Bracon brevicornis* (Wesmael), *Coccygidium luteum* (Brullé), *Coccygidium melleum* (Roman), *Coccygidium* sp., *Coccygidium transcaspicum* (Kokujev), *Glyptapanteles creatonoti* (Viereck), *Meteorus pulchricornis* (Wesmael), *Microplitis demolitor* (Wilkinson), *Snellenius manilae* (Ashmead), *Euplectrus* sp.nr. *xanthocephalus* Girault (Hymenoptera: Bethylidae), *Campoletis chlorideae* (Uchida), *Charops bicolor* (Szepligeti), *Eriborus* sp., *Ichneumon promissorius* (Erichson), *Metopius rufus* Ashmead, *Netelia* sp., *Temelucha* sp. (Hymenoptera: Ichneumonidae), *Exorista sorbillans* (Wiedemann), and *Exorista xanthaspis* (Wiedemann) (Diptera: Tachinidae), and predators include *Polistes* cf. *olivaceus* (De Geer), *Ropalidia brevita* (Das and Gupta) (Hymenoptera: Vespidae), *Cicindela* sp. (Coleoptera: Cicindelidae), *Brumoides sututralis* (F.), *C. sexmaculata*, *Coccinella transversalis* (F.), *Cycloneda sanguinea* (L.), *Harmonia octomaculata* (Fabricius) (Coleoptera: Coccinellidae), *Paederus fuscipes* Curtis (Coleoptera: Staphylinidae), *Forficula* sp. (Dermaptera: Forficulidae), *Orius* sp. (Hemiptera: Anthocoridae), *Andrallus spinidens* (F.), *Eucanthecona furcellata* (Wolff), *Podisus maculiventris* (Say) (Hemiptera: Pentatomidae), *Cosmolestes* sp., *Rhynocoris fuscipes* (F.), *Rhynocoris marginatus* (F.) (Hemiptera: Reduviidae), *Oxyopes birmamicus* Thorell (Araneae: Oxyopidae), *Lycosa* sp. (Araneae.: Lycosidae), *Marpissa* sp., and *Rhene flavicomans* (Simon) (Araneae: Salticidae) [90]. A combination of native and naturalized natural enemies are providing substantial suppression of the fall armyworm.

Palm infesting whitefly, *Aleurotrachelus atratus* Hempel (Hemiptera: Aleyrodidae) is a native of Brazil reported from India in 2019 [91]. It colonizes more than 110 plant species with preference to plants belonging to the family Arecaceae. The parasitoid *E. cubensis*, a native of the neotropics accidentally introduced to India, was reported to parasitize this whitefly [92]. Predators, *A. astur* (Neuroptera: Chrysopidae), *Cybochephalus* spp. (Coleoptera: Cybocephalidae), *C. nigrita* and *J. pallidula* (Coleoptera: Coccinellidae) have been reported [91].

Woolly whitefly, *Aleurothrixus floccosus* (Maskell) (Hemiptera: Aleyrodidae) is a native of the neotropics reported from India in 2019 [93]. It is a polyphagous species, globally known to feed on 20 different plant families with a preference for citrus and guava. Parasitoid, *E. cubensis* has been reported on this whitefly [79]. Predators, *A. astur* (Neuroptera: Chrysopidae), *Acletoxenus indicus* (Malloch) (Diptera: Drosophilidae), *C. montrouzieri* and *Scymnus nr. utilis* (Hoang) (Coleoptera: Coccinellidae) have been reported [94]. These natural enemies are providing substantial control of woolly whitefly.

Cassava mealybug, *Phenacoccus manihoti* (Matile-Ferrero) (Hemiptera: Pseudococcidae), a native of Brazil was introduced to India in 2020 [95]. The parasitoid *Anagyrus lopezi* De Santis (Hymenoptera: Encyrtidae) of South American origin was imported from Benin in 2021 and field released in 2022 [96,97]. Local natural enemies encountered were *Hyperaspis maindroni* (Sicard), *C. sexmaculata*, *Scymnus coccivora* Ramakrishna Ayyar (Coleoptera: Coccinellidae), *S. epius* (Lepidoptera: Lycaenidae), *Stathmopoda* sp. (Lepidoptera: Stathmopodidae), *Nola* sp. (Lepidoptera: Nolidae), *Anatrachyntis* sp. (Lepidoptera: Cosmopterigidae), *Mallada desjardinsi* (Navas), *Aspertochrysa* (=*Pseudomallada*) *astur* (Banks), (Neuroptera: Chrysopidae), *Carpophilus mutilates* (Erichson) (Coleoptera: Nitidulidae), *Cardiastethus* sp. (Hemiptera: Anthocoridae) and *Indoxysticus* sp. (Araneae: Thomisidae) [98]. Introduction of *A. lopezi* immediately after observing the establishment of *P. manihoti* resulted not only in successful control of the mealybug but also curtailing associated economic losses.

Walnut leaf miner, *Caloptilia roscipennella* (Hübner) (Lepidoptera: Gracillariidae), is a leaf-mining moth native to Central and Southern Europe that was recorded from Kashmir, infesting walnut crops and causing leaf damage ranging from 15% to 20% [99]. Several parasitoid species were reared from infested mines, notably of the genus *Chrysocharis* Förster (Hymenoptera: Eulophidae) and *Itoplectis maculator* (Fabricius) (Hymenoptera: Ichneumonidae). These parasitoids provided effective control of the walnut leaf miner, with parasitization rates reaching up to 70%.

Annona whitefly, *Aleurotrachelus anonae* (Corbett) (Hemiptera: Aleyrodidae) of neotropical origin, was reported from Karnataka, India in 2024 [100]. Parasitism by *Encarsia* sp. and *Eretmocerus* sp. (Hymenoptera: Aphelinidae) and predation by *Scymnus latemaculatus* Motschulsky (Coleoptera: Coccinellidae) were reported [100].

### 2.3. Invasive Weeds

Common prickly pear, *O. monocantha*, native to southern Brazil and Uruguay, was introduced to India in the 1780s and it became invasive from Punjab to Assam. Introduction of the cochineal insect *Dactylopius ceylonicus* (Green) (Hemiptera: Dactylopiidae) in 1795 from Brazil resulted in successful control of the weed. It became the first documented record of biological control of an alien invasive plant anywhere in the world [7].

Erect prickly pear, *O. stricta*, originated from Florida and West Indies region, was introduced to India in the mid-1800s. Intentional introduction of *Dactylopius opuntiae* (Cockerell) (Hemiptera: Dactylopiidae) from Sri Lanka in 1926 resulted in its successful control in Southern India [11].

Red-flower prickly pear, *O. elatior* is a native of tropical South America, and it was introduced around 1872 (specimen deposited in the Kew Gardens). In 1926, *D. opuntiae* was intentionally introduced from Sri Lanka for *O. elatior* control, but this also resulted in successful control of *O. stricta* [11].

Lantana, *L camara* is a pantropical weed native to the tropical Americas. It was introduced to India in 1809. Ramachandra Rao (1920) [9] surveyed India and Burma and reported occurrence of 148 local insects on lantana, of which *Lantanophaga pusillidactylus* (Walker) (Lepidoptera: Pterophoridae) and *Aspondylia lantanae* Felt (Diptera: Cecidomyiidae) are fortuitously introduced insects. Muniappan and Viraktamath [101] reported fortuitous establishment of *Epinotia lantana* (Busck) (Lepidoptera: Tortricidae), a native of Mexico in southern India. Exotic natural enemies introduced were *Ophiomyia lantanae* (Froggatt) (Diptera: Agromyzidae) from Hawaii (Origin: Mexico) in 1921, *Teleonemia scrupulosa* Stål (Hemiptera: Tingidae) from Australia in 1941, *Uroplata girardi* Pic. (Coleoptera: Chrysomelidae) in 1969, *Octotoma scabripennis* Guerin (Coleoptera: Chrysomelidae) from Australia in 1971 [101]. These natural enemies have provided only partial control of lantana due to various abiotic and biotic factors.

Crofton weed, *Ageratina adenophora* (Sprengel) (King and Robinson) (Asteraceae) is a native of Central America, and it was introduced to India in the early twentieth century. A gall fly *Procecidochares utilis* (Stone) (Diptera: Tephritidae) from Mexico was introduced to India in 1963 [17]. It has established in hill stations in Tamil Nadu and West Bengal and spread from India to Nepal [102] and even crossed the Himalaya and reached China in 1984 [103]. Despite heavy parasitism by indigenous natural enemies, it has provided partial control of Crofton weed [104].

Siam weed, *C. odorata*, is a native of the neotropics introduced to India in the early 1840s through the botanical garden in Kolkata. A coffee planter Mr. Kalappa in Coorg upon finding *C. odorata* in his estate convinced Government of Karnataka to support a biological control project to manage it. At the request of Government of Karnataka, CIBC initiated a project resulting in the introduction of *Parachaetes pseudoinsulata* (Rego Barros) (Lepidoptera: Erebidae) into Karnataka, India in 1970 from Trinidad [105]. Additionally, a shipment *P. pseudoinsulata* was received from Sri Lanka in 1984, cultured and field released [106]. Sporadic occurrence of *P. pseudoinsulata* and defoliation of *C. odorata* have been reported from Kerala, Karnataka, and Tamil Nadu [107,108]. The eriophyid mite, *Acalitus adoratus* (Keifer) (Acari: Eriophyidae) was fortuitously introduced to India in 2000 [109]. The gall fly *Cecidochares connexa* (Macquart) (Diptera: Tephritidae) was introduced into Karnataka in 2002 from Indonesia [110]. Later it was introduced to Kerala and Chhattisgarh [111]. *Orimyrus orientalis* (Walker) (Hymenoptera: Ormyridae) has been found to parasitize *C. connexa* at moderate to high levels [112]. *Parachaetes pseudoinsulata* and *C. connexa* have partially suppressed the Siam weed.

Parthenium, *Parthenium hysterophorus* L. (Asteraceae), is a native of Mexico, introduced to India in the 1950s. The natural enemy *Calligrapha* (=*Zygogramma*) *bicolorata* (Pallister) (Coleoptera: Chrysomelidae) was introduced to Bengaluru in 1984 [113]. It has spread all over India and moved to neighboring countries Bangladesh, Bhutan, Nepal, Pakistan, and Sri Lanka. Fortuitously introduced Winter rust *Puccinia abrupta* var. *partheniicola* (Pucciniaceae) has been reported to occur sporadically in southern India from the 1980s [114]. *Calligrapha bicolorata* has substantially controlled parthenium, however, introduction of additional natural enemies should be considered for effective suppression.

Water hyacinth, *Pontederia* (=*Eichhornia*) *crassipes* (Pontederiaceae) is a native of Brazil and it was introduced to Calcutta Botanical Gardens in 1890 as an ornamental plant. Currently, it has spread to all water bodies and rivers in India [115]. Three natural enemies *Neochetina bruchi* Hustache, *Neochetina eichhorniae* (Warner) (Coleoptera: Curculionidae) and *Orthogalumna terebrantis* Wallwork (Acarina: Galumnidae) of South American origin were introduced to India from USA [116]. The mite and weevils were released in 1982 and 1983, respectively. These natural enemies have partially suppressed water hyacinth.

Water fern, *S. molesta*, is an aquatic weed, and it is a native of south-eastern Brazil [117]. It was first observed in the 1955 in Vali Lake, Trivandrum, Kerala in India [15,118]. A grasshopper, *Paulinia acuminata* De Geer (Orthoptera: Acrididae) was introduced from Trinidad in 1974 but was not effective [117,119]. The weevil *Cyrtobagous salviniae* (Calder and Sands) (Coleoptera: Curculionidae) of Brazilian origin was imported from Australia to Bengaluru in 1982 and field released in Kerala, Jammu and Kashmir, Bhubaneshwar, and Hyderabad in 1983 [15]. Further releases of *C. salviniae* were made in Padua village of Katni district in December 2019 [120] and Satpura water reservoir at Sarni in October 2022 [121] in Madhya Pradesh; Chandpur and Gadchiroli districts in Maharashtra in January 2022 [122]; and Tulpuri lake in Durg district, Chhattisgarh [120]. Excellent control of *S. molesta* was achieved by introducing *C. salviniae*.

Water lettuce, *Pistia stratioides* L. (Araceae), has unknown origin, and it has been speculated to be from Africa, Asia, or South America. It is a monocotyledonous aquatic weed and occurs in water bodies and rivers all over India. A local insect, *Spodoptera pectinicornis* (Hampson) (Lepidoptera: Noctuidae), native to South Asia, has been reported to cause extensive damage to this weed in Kerala, India [27]. It has been mass-reared and released in the fields for control of this weed in Thailand [123]. In 1982, a weevil *Neohydronomus affinis* (Hustache) (Coleoptera: Curculionidae) was introduced to Australia from Brazil and it has provided effective control [124]. India may introduce *N. affinis* if *P. stratioides* is still considered as a problem.

Mile-a-minute vine, *Mikania micrantha* (Kunth) (Asteraceae), is a native of Caribbean islands introduced through Kew to Kolkata botanical garden in the early 1900s [125]. A rust fungus *Puccinia spegazzinii* (Uredinales: Pucciniaceae) was released in Assam and Kerala in July 2005 but failed to establish even though it established in Taiwan, Papua New Guinea, and Fiji [126,127].

Giant sensitive plant, *Mimosa diplotricha* C. (Wright ex Saivalle) (Mimosaceae), is a native of Central America to Brazil and it has been reported from Kerala, Tamil Nadu, Karnataka, Assam, Odisha, Uttar Pradesh, Nagaland, and Tripura in India [128,129]. No classical biological control action has been taken on this weed in India. It has been successfully controlled with the introduction of a natural enemy *Heteropsylla spinulosa* (Muddiman, Hodkinson and Hollis) (Hemiptera: Psyllidae) in some Pacific Island countries and Australia [130].

### 2.4. Classical Biological Control

Successful control of the invasive weeds includes control of *O. monocantha* in northern India by the serendipitous introduction of *D. ceylonicus* in 1795 led to the idea of using insects for control of weeds [7]. Subsequently, control of the cactuses, *O. stricta* and *O. elatior* in southern India was achieved by the intentional introduction of *D. opuntiae* from Sri Lanka in 1926 [11]. The invasive water fern, *S. molesta* was successfully controlled in the states of Kerala, Chhattisgarh, Madhya Pradesh, and Maharashtra by the introduction of the weevil *C. salviniae* from Australia in 1982 [118]. Substantial control of the weed parthenium *P. hysterophorus* has been achieved by introducing *C. bicolorata* [131]. Only partial control of the weeds, *A. adinophora*, *C. odorata* and *P. crassipes* have been achieved.

Invasive pests of temperate fruit crops, woolly apple aphid *E. lanigerum*, and San Jose scale *C. perniciosa* are under control by the introduced natural enemies, but there is a need for inoculative release of their natural enemies early in the spring to catch up with the host population that emerges from diapause. Invasive pests of tropical crops, coffee green scale *C. viridis*, cottony cushion scale *I. purchasi*, Leucaena psyllid *H. cubana*, citrus mealybug *P. citri*, eucalyptus gall wasp *L. invasa*, papaya mealybug *P. marginatus*, and cassava mealybug *P. manihoti*, are successfully controlled by the intentionally introduced natural enemies requiring minimal or no additional efforts [15,45,54,67]. Successful control of cotton mealybug *P. solenopsis* has been achieved by the fortuitously introduced parasitoid *A. arizonensis* [64]. Additionally, accidentally introduced parasitoids *E. cubensis*, *E. dispersa*, *E. guadeloupae*, and *E. haitiensis* have been providing effective control of invasive whiteflies spiralling whitefly *A. dispersus*, and rugose spiralling whitefly *A. rugioperculatus*, and substantial control of solanum whitefly *A. trachoides*, and woolly whitefly *A. floccosus* [48,79,83,84,132]. Substantial control of potato tuber moth *P. operculella*, and diamondback moth, *P. xylostella* were achieved with the introduced natural enemies [22,26,28,31]. Palm infesting whitefly *A. atratus*, Madeira mealybug *P. madeirensis* and Jack Beardsley mealybug *P. jackbeardsleyi* are substantially controlled by accidentally introduced and native natural enemies [69,72,79,92]. There has always been varying degrees of association between local natural enemies and introduced natural enemies in managing invasive insect pests. Number of natural enemies intentionally and fortuitously introduced and local natural enemies attacking various invasive insect pests and weeds are given in Table 1.

Within India, parasitoids, *E. flavoscutellum* and *O. pallidipes intentionally* transferred from the northeastern region to the southern region for control of sugarcane woolly aphid, *C. lanigera* and banana skipper, *E. torus*, respectively, have given satisfactory control of both pests [60,74].

Few of the introduced natural enemies have extended their host range beyond their intended target(s) controlling additional introduced exotic pests. The coccinellid beetle *C. montrouzieri* introduced for control of coffee green scale *C. viridis* is also providing control of several introduced and native mealybugs and scale insects, spherical mealybug *Nipaecoccus viridis* (Newstead), hibiscus mealybug *Maconellicoccus hirsutus* (Green), coffee mealybug *Planococcus lilacinus* (Cockerell), striped mealybug *Ferrisia virgata* Cockerell, mango mealybug *Rastrococcus iceryoides* (Green), fruit tree mealybug *Rastrococcus invadens* Williams, eggplant mealybug *Coccidohystrix insolita* (Green), passionvine mealybug *Planococcus minor* (Maskell), longtailed mealybug *Pseudococcus longispinus* (Targioni Tozzetti) (Hemiptera: Pseudococcidae), green shield scale *Pulvinaria psidii* (Maskell), wax scale *Drepanococcus chiton* (Green), cottony citrus scale *Pulvinaria polygonata* (Cockerell), hemispherical scale *Saissetia coffeae* (Walker), *Fistulococcus pokfulamensis* (Hodgson and Martin) (Hemiptera: Coccidae) and *Uhleria araucariae* (Maskell) (Hemiptera: Eriococcidae) [15,133]. An exotic parasitoid *T. remus*, introduced for control of *S. litura* and *A. janata* in 1963 [17], has proven to be one of the effective agents for controlling fall armyworm *S. frugiperda* that invaded India in 2018 [90]. Exotic parasitoids introduced, *T. evanescens* for stem borers, and *T. brasiliensis* and *T. pretiosum* for *Helicoverpa* sp. (Lepidoptera: Noctuidae) have been reported to parasitize diamondback moth *P. xylostella* in India [28,33].

#### 2.4.1. Successes

Until 2004, a total of 166 exotic natural enemies of insect pests and weeds were imported and studied in India. Out of these, 71 species established in the field resulting in six, excellent; seven, substantial; and four, partial control of invasive insect pests and weeds [15]. By 2018, a total of 22 species of natural enemies of weeds established in the field, of which, seven, four, and nine agents provided excellent, substantial, and partial control, respectively [134]. Since 2004, eight exotic natural enemies were imported, of which six established in the field, one did not establish, and one has yet to be released in the field. We found either intentionally or fortuitously introduced natural enemies provided fifteen, excellent; nine, substantial; and four, partial control of invasive insect pests and weeds (Table 2).

#### 2.4.2. Constraints

Biotic constraints such as local hyper parasitoids attacking introduced parasitoids; and parasitoids attacking predators of invasive insect pests and introduced natural enemies of invasive weeds have reduced efficacy of the introduced natural enemies. The parasitoid, *A. arizonensis* of cotton mealybug *P. solenopsis* is attacked by hyper parasitoids *Promuscidea unfasciativentris* (Girault), *Myiocnema comperei* (Ashmead), *Prochiloneurus albifuniculus* (Hayat, Alam and Agarwal), *Prochiloneurus pulchellus* (Silvestri), *Prochiloneurus aegyptiacus* (Mercet) (Hymenoptera: Encyrtidae) and *Marietta leopardina* (Motschulsky) (Hymenoptera: Aphelinidae) [64]. *Lantana camara* natural enemies, *O. lantanae* is attacked by eight species of chalcidoid parasitoids ([135] and *T. scrupulosa* by an egg parasitoid, *Erythmelus teleonemiae* (Subba Rao) (Hymenoptera: Mymaridae) [136]. Four parasitoids, *Diameromicrus kiesenwetteri* (Meyr) (Hymenoptera: Torymidae), *Syntomopus* sp. (Hymenoptera: Pteromalidae), *Bracon* sp. (Hymenoptera: Braconidae), and *Eurytoma* sp. (Hymenoptera: Eurytomidae) identified on *P. utilis* impacted its efficacy on control of Crofton weed *A. adenophora* [104]. The parasitoid, *Ormyrus orientalis* Walker (Hymenoptera: Ormyridae) has been observed to attack *C. connexa*, the natural enemy of Siam weed *C. odorata* in Bengaluru [112]. Parasitoids *Aprostocetus* sp. (Hymenoptera: Eulophidae), *Homalotylus turkmenicus* (Myartseva), *H. flaminus* Dalman (Hymenoptera: Encyrtidae), and *Metastenus concinnus* (Walker) (Hymenoptera: Pteromalidae) attack immature stages of the ladybeetle *Hyperaspis maindroni* (Sicard) (Coleoptera: Coccinellidae). *Tetrastichus* sp. (Hymenoptera: Eulophidae) and *Brachycyrtus* sp. (Hymenoptera: Ichneumonidae) parasitize larvae of *Mallada desjardinsi* (Navas) (Neuroptera: Chrysopidae) [69]. The parasitoid *Palexorista* sp. (Diptera: Tachinidae) and predators, *Andrallus spinidens* (F), *Eocanthecona furcellata* (Wolff) (Hemiptera: Pentatomidae) and *Sycanus pyrrhomelas* (Walker) (Hemiptera: Reduviidae), were reducing the population of the leaf feeding beetle *C. bicolorata* introduced for control of parthenium [137].

#### 2.4.3. Gaps

There are gaps in implementing classical biological control of introduced insect pests and weeds. For example, invasive insects *I. insignis* and *Q. erythrinae* were effectively controlled by introduced natural enemies in other countries [35,36,70] and India could consider introducing them. Similarly, effective natural enemies used to control invasive weeds *M. diplotricha* and *P*. *stratioides* in other countries could be considered for introduction [124,130]. Efforts made to manage *M. micrantha* by introducing the rust fungus should be followed up [127]. Some agents introduced for control of invasive weeds are yet to be distributed widely in India. For example, *Octotoma scabripennis* was established in northern India in 1971 but has not yet been introduced to southern India. Furthermore, *C. connexa*, established in the Bengaluru area in 2002, has not been released in northeastern India, where *C. odorata* is a serious problem. While addressing biological control of invasive insect pests has been streamlined, the same for invasive weeds requires strengthening.

#### 2.4.4. Impact Assessment

Only a few reports are available on assessing economic impact of controlling invasive insect pests and weeds by introduced exotic natural enemies in India. Myrick et. al. [138] estimated that India benefited from USD 540 million to USD 1.34 billion by introducing the parasitoid *A. papayae* for control of papaya mealybug *P. marginatus*. According to Selvaraj et. al. [139] adoption of biological control strategies for management of rugose spiralling whitefly on coconut and oil palm plantations resulting in a saving of Indian Rs 9500/ha. Ballal [16] reported release of *C. salviniae* for control of *S. molesta* resulting in savings (on labor alone) of Indian Rs 6.9 million annually. Impact assessment is essential to understand the benefits of the projects as well as to gain support of donors and policy makers.

### 2.5. Augmentative Biological Control

Trichogrammatids are the most used agents in inundated augmentative biological control. According to Jalali [33], there are 19 introduced exotic species and 32 locally identified species of *Trichogramma* in India of which, *T. chilonis*, *T. japonicum*, *T. achaeae*, *T. embryophagum*, *T. pretiosum*, *T. brasiliensis*, and *T. bactrae* are commonly used in augmentative control, mostly for the lepidopteran pests. To cite a few examples, Krishnamoorthy et al. [140] recommended release of either one of the local parasitoids *T. bactrae*, *T. chilonis* or the exotic parasitoid *T. brassicae* at the rate of 50,000 adults per hectare per week for the effective control of *P. xylostella*. Singh [28] recommended release of *T. embryophagum* at 2000 adults/tree at weekly intervals, starting when the first moth is caught in the pheromone trap for codling moth *L. pomonella* management. Additionally, Singh [28] has given a list for augmentative releases of *T. brasiliensis*, *T. chilonis*, *T. pretiosum*, *A. mali*, *L. dactylopii*, *N. cardinalis*, *C. montrouzieri*, and *Phytoseiulus persimilis* Athias-Henriot (Acari: Phytoseiidae) and their dosages for control of various vegetable and fruit crop insect and mite pests in India. However, India lags far behind European countries in commercial production and utilization of natural enemies for augmentative control especially in macrobials.

### 2.6. Conservation Biological Control

Conservation biological control plays a major role in successes of classical and augmentative biological control by enhancing the efficacy of natural enemies. Indiscriminate pesticide use is a major negative factor in conservation biological control [141]; however, pesticide use in India is low at 0.4 kg/ha compared to 1.83 kg/ha in China. Additionally, pesticide use has declined from 0.44 kg/ha in 1990 to 0.37 kg/ha by 2021 [142] due to implementation of integrated pest management (IPM). There are 36 Central Integrated Pest Management Centers (CIPMC) located in 28 states and two union territories are promoting the use of biopesticides and natural enemies and emphasizing conservation biological control. It is a positive and encouraging development, which strengthens conservation biological control and enhances environmental safety in India.

There are several instances of successful conservation biological control due to creation of safer environment for natural enemies. *Cryptolaemus montrouzieri* introduced for management of *C. viridis* provided control of several mealybugs and scale insects [15]. Native and naturalized natural enemies successfully managed South American tomato leaf miner, *P. absoluta*, banana skipper *E. torus*, American serpentine leafminer *L. trifolii*, Jack Beardsley mealybug *P. jackbeardsleyi*, Madeira mealybug *P. madeirensis*, fall armyworm *S. frugiperda*, and some of the recently introduced whiteflies.

## 3. Way Forward

Even though varying degrees of success in managing invasive insect pests and weeds has been achieved by adopting biological control, it would be worthwhile to explore natural enemies of some of the exotic pests of economic and environmental importance, such as mango fruit borer *Citripestis eutraphera* (Meyrick) (Lepidoptera: Pyralidae), red banded mango caterpillar *Deanolis sublimbalis* Snellen (Crambidae: Lepidoptera), tobacco thrips *T. parvispinus*, western flower thrips *Frankliniella occidentalis* (Pergande) (Thysanoptera: Thripidae), legume feeding whitefly *Tetraleurodes acaciae* (Quaintance) (Hemiptera: Aleyrodidae), Bondar’s nesting whitefly *Paraleyrodes bondari* Peracchi (Hemiptera: Aleyrodidae), apple leaf blotch miner *Leucoptera malifoliella* (O. Costa) (Lepidoptera: Lyonetiidae), cactus mealybug *Hypogeococcus pungens* (Granara de Willink) (Hemiptera: Pseudococcidae), pit scale *Hyalococcus striatus* (Russell) (Hemiptera: Asterolecaniidae), mango soft scale *Fistulococcus pokfulamensis* (Hodgson and Martin) (Hemiptera: Coccidae), and others that have already established in India. When no effective natural enemies are found, effective alternate IPM components should be explored and implemented.

An invasive insect pest of coconut trees and a native of Southeast Asia, the hispine beetle *Brontispa longissima* (Gestro) (Coleoptera: Chrysomelidae), has established in the neighboring countries Maldives and Myanmar [143,144]. As such, India should take necessary quarantine measures to prevent its introduction and to take appropriate control measures when it invades. One of the options available is the introduction of the parasitoids *Asecodes hispinarum* Boucĕk and *Tetrastichus brontispae* (Ferrière) (Hymenoptera: Eulophidae) that have had successful results in several Asia–Pacific countries [144]. Giant sensitive tree, *Mimosa pigra* L. (Leguminosae), an invasive weed native to the neotropics, has established in Myanmar and Sri Lanka [145]. The report of its occurrence in India by Welgama et al. [146] is an error. It also requires quarantine measures to prevent introduction and appropriate control measures to be taken when it gets introduced.

## 4. Conclusions

The first documented biological control of a weed took place in India in 1795, leading to the initiation of biological control programs around the world. However, until the start of the Project Directorate of Biological control in 1977 (presently, ICAR–National Bureau of Agricultural Insect Resources), most of the activities were sporadic and lacked systematic implementation and follow up. Successful control was achieved of the weeds, *O. monacantha*, *O. stricta*, *O. elatior*, and *S. molesta*, and insect pests, *A. dispersus*, *C. viridis*, *A. rugioperculatus*, *C. perniciosa*, *H. cubana*, *I. purchasi*, *L. invasa*, *P. marginatus*, *P. manihoti*, and *P. solenopsis*, by adopting classical biological control. The coccinellid beetle, *C. montrouzieri*, introduced from Australia in 1898, has contributed to control of several species of mealybugs and scale insects. Partial control was achieved of *L. camara*, *C. odorata*, *E. lanigerum*, *A. atratus*, *A. floccosus*, and *P. hysterophorus*. Augmentative control was adopted using both exotic and local natural enemies for *E. lanigerum*, *P. opercullela*, *P. xylostella*, *L. pomonella*, *P. absoluta*, and *S. frugiperda*. Efficacy of some of the introduced natural enemies of weeds was reduced by the local parasitoids, predators of insect pests by parasitoids, and parasitoids by hyperparasitoids. In general, India has benefited to the tune of billions of dollars by managing invasive species and safeguarding the environment through adaptation of biological control programs. The declining trend in use of chemical pesticides in recent years is a step in the right direction for sustainable development. With the development of human resources in employing cutting-edge technologies, such as molecular tools, artificial intelligence, and machine learning, biological control of invasive species activities could be substantially improved.

## Figures and Tables

**Table 1:** Intentionally and fortuitously introduced and local natural enemies attacking various invasive insect pests and weeds.

Invasive Insect Pests and Weeds	Country/Region of Origin	Year of Introduction to India	Year of First Natural Enemy Introduced	No. of Exotic Natural Enemies Introduced and Established	No. of Fortuitously Introduced Natural Enemies	Local Natural Enemies or Ones Introduced for Other Pests
*Eriosoma lanigerum*	China	1889	1930	1		11
*Coccus viridis*	Brazil	1890s	1898	1		8
*Comstockaspis perniciousa*	China	1910	1960	4		7
*Phthorimaea operculella*	South America via Italy	1906	1944	17		1
*Plutella xylostella*	Europe/South Africa	1914	1994	3	3	3
*Icerya purchasi*	Australia	1928	1929	1		2
*Laspeyresia pomonella*	Europe and Northwestern Asia	1972	1993	1		3
*Paracoccus marginatus*	Mexico	2007	2010	3		7
*Heteropsylla cubana*	Central America	1988	1988	1		2
*Hypothenemus hampei*	West Africa	1990	1995	3		
*Quadrastichus erythrinae*	Africa	2005				1
*Phenacoccus solenopsis*	USA	2006	2009		1	19
*Leptocybe invasa*	Australia	2001	2008	1		3
*Phthorimaea absoluta*	South America	2014				7
*Liriomyza trifolii*	Florida, USA	1990	1997	1 (did not establish)		2
*Ceratovacuna lanigera*	Southeast Asia	1958—West Bengal. 2002—Maharashtra and southern India	-	-	-	4
*Aleurodicus dispersus*	Central America and the Caribbean region	1994	-	-	2	13
*Aleurodicus rugioperculatus*	Central America	2016	-	-	2	6
*Aleurotrachelus trachoides*	Neotropical	2014	-	-	1	-
*Tetraleurodes acaciae*	Central America and the Caribbean	2017	-	-	1	-
*Paraleyrodes bondari*	Neotropical	2017	-	-	-	3
*Spodoptera frugiperda*	Americas	2018	-	-	-	46
*Aleurotrachelus atratus*	Brazil	2019	-	-	1	4
*Aleurothrixus floccosus*	Neotropics	2019	-	-	1	4
*Aleurotrachelus anonae*	Neotropics	2024	-	-	2	1
*Erionota torus*	Northern India to Southern China	2012 in Southern India	-	-	-	14
*Phenacoccus manihoti*	South America	2020	2022	1	-	12
*Phenacoccus madeirensis*	Neotropics	2012	-	-		17
*Pseudococcus jackbeardsleyi*	Neotropics	2012	-	-	-	7
*Ageratina adenophora*	Central America	Early 20th century	1963	1	-	-
*Chroromolaena odorata*	Neotropics	Early 1840s	1972	2	1	-
*Lantana camara*	Tropical America	1809	1921	4	3	1
*Opuntia monocantha*	South America	1700s	1795	1	-	-
*Opuntia stricta*	North America	Mid-1800	1926	1		-
*Opuntia elatior*	South America	1872	1926	1	-	-
*Parthenium hysterophorus*	Neotropics	1950s	1983	1	1	-
*Pistia stratiotes*	Southeastern United States			-	-	1
*Pontederia crassipes*	South America	Before 1900	1982	3	-	-
*Salvinia molesta*	Brazil	1955	1982	2	-	-

**Table 2:** Invasive species of insects and weeds suppressed by intentionally or fortuitously introduced exotic natural enemies in India.

Number	Excellent	Substantial	Partial
1	*Aleurodicus dispersus* Russell (Hemiptera: Aleyrodidae)	*Aleurotrachelus trachoides* (Back) (Hemiptera: Aleyrodidae)	*Ageratina adenophora* (Spreng.) King and H. Rob. (Asteraceae)
2	*Aleurodicus rugioperculatus* Martin (Hemiptera: Aleyrodidae)	*Aleurothrixus floccosus* (Maskell) (Hemiptera: Aleyrodidae)	*Chromolaena odorata* (L.) R. M. King and H. Rob. (Asterales: Asteraceae)
3	*Coccus viridis* (Green) (Hemiptera: Coccidae)	*Eriosoma lanigerum* (Hausmann) (Hemiptera: Aphididae)	*Lantana camara* L. (Verbenaceae)
4	*Comstockaspis perniciosa* (Comstock) (Hemiptera: Diaspididae)	*Parthenium hysterophorus* L. (Asteraceae)	*Pontederia crassipes* Mart. (Pontederiaceae)
5	*Heteropsylla cubana* (Crawford) (Hemiptera: Psyllidae)	*Phenacoccus madeirensis* (Green) (Hemiptera: Pseudococcidae)	
6	*Icerya purchasi* Maskell (Hemiptera: Monophlebidae)	*Phthorimaea operculella* (Zeller) (Lepidoptera: Gelechiidae)	
7	*Leptocybe invasa* (Fisher and La Salle) (Hymenoptera: Eulophidae)	*Plutella xylostella* (Linnaeus) (Lepidoptera: Plutellidae)	
8	*Paracoccus marginatus* (Williams and Granara de Willink) (Hemiptera: Pseudococcidae)	*Pseudococcus jackbeardsleyi* (Gimpel and Miller) (Hemiptera: Pseudococcidae)	
9	*Planococcus citri* (Risso) (Hemiptera: Pseudococcidae)	*Spodoptera frugiperda* (J.E. Smith) (Lepidoptera: Noctuidae)	
10	*Phenacoccus manihoti* (Matile-Ferrero) (Hemiptera: Pseudococcidae)		
11	*Phenacoccus solenopsis* Tinsley (Hemiptera: Pseudococcidae)		
12	*Opuntia elatior* (Miller) (Cactaceae)		
13	*Opuntia monacantha* (Haw.) (Cactaceae)		
14	*Opuntia stricta* (Haw.) (Cactaceae)		
15	*Salvinia molesta* (S.D. Mitchel) (Salviniaceae)		

## Data Availability

No new data were created or analyzed in this study. Data sharing is not applicable to this article.

## Supplementary Materials

The following supporting information can be downloaded at: https://www.mdpi.com/article/10.3390/insects17010061/s1, Table S1. List of invasive insect pests recorded in the past 20 years.

## References

[1] Ramakrishnan P.S. (1991). Ecology of Biological Invasion in the Tropics.

[2] Bang A., Cuthbert R.N., Haubrock P.J., Fernandez R.D., Moodley D., Diagne C., Turbelin A.J., Renault D., Dalu T., Courchamp F. (2022). Massive economic costs of biological invasions despite widespread knowledge gaps: A dual setback for India. Biol. Invasions.

[3] Muniappan R., Reddy G.V.P., Raman A. (2009). Biological Control of Tropical Weeds Using Arthropods.

[4] Sushil S.N., Sampathkumar M., Shylesha A.N., Subaharan K. (2024). Invasive Insect Pest Threats to Indian Agriculture: Present status, Challenges and Way Forward. Indian. J. Plant Prot..

[5] Ehler L.E., Barbosa P. (1998). Conservation biological control: Past, present, and future. Conservation Biological Control.

[6] Ferro D.N., McNeil J.N., Barbosa P. (1998). Habitat enhancement and conservation of natural enemies of insects. Conservation Biological Control.

[7] Zimmermann H., Moran C., Hoffmann J., Muniappan R., Reddy G.V.P., Raman A. (2009). Invasive cactus species (Cactaceae). Biological Control of Tropical Weeds Using Arthropods.

[8] Goeden R.D. (1988). A capsule history of biological control of weeds. Biocontrol News Info..

[9] Ramachandra Rao Y. (1920). Lantana insects in India. Mem. Dep. Agric. India Entomol. Ser..

[10] Subramanyam T.V. (1934). The Lantana seed fly in India, *Agromyza* (*Ophiomyia*) *lantanae* Froggatt. Indian J. Agric. Sci..

[11] Kunhikannan K. (1928). The introduction of a new insect into Mysore (India). Agric. J..

[12] Muniappan R. Opportunities and constraints for classical weed biocontrol in developing countries. Proceedings of the XV International Symposium on Biological Control of Weeds.

[13] Sushilkumar, Anokhe A., Singh N., Deeksha M.G. (2024). Climate change effect on the efficacy of biological control agents of terrestrial and aquatic invasive weeds. Indian J. Weed Sci..

[14] Mayne W.W. (1953). *Cryptolaemus montrouzieri* Mulsant in South India. Nature.

[15] Singh S.P. (2004). Some Success Stories in Classical Biological Control of Agricultural Pests in India.

[16] Ballal C. (2022). Success stories in biological control: Lessons learnt. Vantage: J. Themat. Anal..

[17] Rao V.P., Ghani M.A., Sankaran T., Mathur K.C. (1971). A Review of the Biological Control of Insects and Other Pests in South-East Asia and the Pacific Region.

[18] Singh S.P. (1989). Achievements of AICRP on Biological Control.

[19] Mathew I.L., Singh D. (2022). Biological control: The origins and the Indian scenario. Intern. J. Entomol. Res..

[20] Asante S.K. (1997). Natural enemies of the woolly apple aphid, *Eriosoma lanigerum* (Hausmann) (Hemiptera: Aphididae): A review of the world literature. Plant Prot. Quart..

[21] Rasool I., Lone G.M., Wani A.R., Pathania S.S., Sharma M.K., Khan N.A., Nazir N., Hussain S. (2019). Natural Enemies fauna associated with woolly apple aphid *Eriosoma lanigerum* Hausmann in Kashmir. J. Entomol. Zool. Stud..

[22] Singh S., Sharma J.H., Udikeri A., Ansari H., El-Shafie H.A.F. (2020). Invasive insects in India. Invasive Species—Introduction Pathways, Economic Impacts, and Possible Management Options.

[23] Puttarudriah M., Channabasavanna G.P., Krishna Murti B. (1952). Discovery of *Cryptolaemus montrouzieri* Mulsant (coccinellidae, Coleoptera, Insecta) in Bangalore, South India. Nature.

[24] Srinivasa M.V. (1987). New parasites and host plants of coffee green scale (*Coccus viridis* (Green)) Homoptera: Coccidae in south India. J. Coffee Res..

[25] Lefroy H.M. (1907). The potato tuber moth. Indian Agric. J..

[26] Chandel R.S., Vashisth S., Soni S., Kumar R., Kumar V. (2020). The Potato Tuber Moth, *Phthorimaea operculella* (Zeller) in India: Biology, Ecology, and Control. Potato Res..

[27] Sankaran T. (1974). Natural enemies introduced in recent years for biological control of agricultural pests in India. Indian J. Agric. Sci..

[28] Singh S.P. (1994). Fifteen Years of AICRP on Biological Control.

[29] Nagarkatti S., Singh S.P. (1989). Importation and establishment of new natural enemies of *Heliothis* spp. (Lepidoptera: Noctuidae) into India. Proceedings of the Workshop on Biological Control of Heliothis: Increasing the Effectiveness of Natural Enemies.

[30] Fotidar M.R. (1941). The San Jose scale and its control in Kashmir. Indian Farming.

[31] Gupta R.P. (2005). Biological control of San Jose scale in India—An overview. Acta Hortic..

[32] Fletcher T.B. (1914). Some South Indian Insects and Other Animals of Importance Considered Especially from an Economic Point of View.

[33] Jalali S.K., Sithanantham S., Ballal C.R., Jalali S.K., Bakthavatsalam N. (2013). Natural occurrence, host range and distribution of trichogrammatid egg parasitoids. Biological Control of Insect Pests Using Egg Parasitoids.

[34] Booth R.G., Cross A.E., Flower S.V., Shaw R.H. (1995). The biology and taxonomy of *Hyperaspis pantherina* (Coleoptera: Coccinellidae) and the classical biological control of its prey, *Orthezia insignis* (Homoptera: Ortheziidae). Bull. Entomol. Res..

[35] Fowler S.V. (2004). Biological control of an exotic scale, *Orthezia insignis* Browne (Homoptera: Ortheziidae), saves the endemic gumwood tree, *Commidendrum robustrum* (Roxb.) DC. (Asteraceae) on the island of St. Helena. Biol. Control.

[36] Ramachandra Rao Y., Cherian M.C. (1944). The fluted scale, *Icerya purchasi* Mask., as a pest of wattle in south India, and its control by biological method. Madras Agric. J..

[37] Patel N.M., Patel N.B., Raghunandan B.L. (2022). Report of *Novius fumidus* Mulsant (Coccinellidae: Coleoptera); A potential predator of Egyptian cottony cushion Scale (*Icerya aegyptiaca* Douglas) infesting *Casuarina equisetifolia* in Gujarat, India. Biol. Forum-Intern. J..

[38] Malik R.A., Punjabi A.A., Bhat A.A. (1972). Survey study of insect and non-insect pests in Kashmir. Horticulture.

[39] (2018). Opinion and Editorial. Scourge of Codling Moth in Ladakh and Its Management. Greater Kashmir.

[40] Kaushik H.D., Arora R.K., Hassan S.A. (1998). *Trichogramma*: Research and use in India. Proceedings of the 5th International Symposium on Trichogramma and Other Egg Parasitoids.

[41] Bindra O.S., Bakhetia D.R.C. (1970). The chemical control of the peach stem aphid, *Pterochlorus persicae* (Cholodkovsky). J. Res. Punjab Agric. Univ..

[42] Mahendiran G., Akbar S.A., Dar M.A. (2018). The invasive aphid *Pterochloroides persicae* (Cholodkovsky, 1899) (Hemiptera: Aphidoidea: Lachninae) recorded on important fruit trees in Kashmir valley, India. J. Threat. Taxa.

[43] Mani M., Thontadarya T.S. (1987). Record of mealybug species on grapevine in Karnataka. Curr. Sci..

[44] Gopalan M., Jayaraj S., Ariavanam M., Pillai K., Subba Rao P.V. (1988). New record of *Heteropsylla cubana* Crawford (Psyllidae: Homoptera) on soobabul *Leucaena leucocephala* (Lam.) de Wit in India. Curr. Sci..

[45] Jalali S.K., Singh S.P. (1989). Release and recovery of an exotic coccinellid predator, *Curinus coeruleus* (Muls.) on subabul psyllid, *Heteropsylla cubana* Crawf. in India. J. Insect Sci..

[46] Rajagopal D., Naik S., Munegowda M.K. (1990). Subabul psyllid and its outbreak in Karnataka. Curr. Res..

[47] Viraktamath C.A., Tiwari G.C., Srinivasan K., Gupta M. (1993). American serpentine leaf miner is new threat to crops. Indian Farming.

[48] Viraktamath C.A. (2002). Alien invasive insect and mite pests and weeds in India and their management. Micrones. Suppl..

[49] Durairaj C. (2007). Bio-Ecology and IPM for Serpentine Leafminer, Liriomyza trifolii Burgess with Special Reference to Cowpea and Tomato.

[50] Kumar P.K.V., Prakasan C.B., Vijayalakshmi C.K. (1990). Coffee berry borer *Hypothenemus hampei* (Coleoptera: Scolytidae): First record from India. J. Coffee Res..

[51] Feriva S.A., Central Coffee Research Institute (2002). Integrated Management of Coffee Berry Borer.

[52] David B.V., Regu K. (1995). *Aleurodicus dispersus* Russell (Aleyrodidae: Homoptera) a whitefly pest, new to India. Pestology.

[53] Mani M., Krishnamoorthy A. (2002). Classical biological control of the spiralling whitefly, *Aleurodicus dispersus* Russell—An appraisal. Insect Sci. Applic..

[54] Yousuf M., Singh S., Ikram M., Singh R.B. (2017). An overview on outbreak of Eucalyptus gall wasp, *Leptocybe invasa* (Hymenoptera: Eulophidae) in Northern India. J. Entomol. Zool. Stud..

[55] Ramanagouda S.H., Vastrad A.S., Narendran T.C., Basavngoud K., Virktamath S. (2011). Current status of eucalyptus gall wasp and its native parasitoids in Karnataka. J. Biol. Control.

[56] Shylesha A.N. (2008). Final Project Report: Classical Biological Control of Eucatyptus Gall Wasp Leptocybe invasa Fisher & La Salle.

[57] Shylesha A.N., Kumar P.S., Sreedevi K., Ballal C.R. (2018). Biocontrol Rescues the Paper Industry: Management of Eucalyptus Gall Wasp, Leptocybe invasa.

[58] Basu A.N., Banerjee S.N. (1958). Aphids of economic plants of West Bengal. Indian Agric..

[59] Baitha A., Triparthi G.M., Singh M.R., Roy S. (2019). Status of invasive woolly aphid management on sugarcane in India. Hexapoda.

[60] Srikanth J., Singaravelu B., Kurup N.K., Mukunthan N., Santhalakshmi G., Nirmala R. (2015). Predators as natural and applied biocontrol agents of sugarcane woolly aphid *Ceratovacuna lanigera* in India: An appraisal. J. Sugarcane Res..

[61] Faizal M.H., Prathapan K.D., Anith K.N., Mary C.A., Lekha M., Rini C.R. (2006). Erythrina gall wasp *Quadrastichus erythrinae*, yet another invasive pest new to India. Curr. Sci..

[62] Kaufman L., Yalemar J., Wright M.G. (2020). Classical biological control of the erythrina gall wasp, *Quadrastichus erythrinae*, in Hawaii: Conserving an endangered Habitat. Biol. Control.

[63] Suresh S., Kavitha P.C., Branco M., Franco J.C., Hodgson C.J. (2008). New records of Coccoidea in India, p. 155. Proceedings of the XI International Symposium on Scale Insect Studies.

[64] Ram P., Saini R.K. (2010). Biological control of solenopsis mealybug, *Phenacoccus solenopsis* Tinsley on cotton: A typical example of fortuitous biological control. J. Biol. Control.

[65] Fand B.B., Suroshe S.S. (2015). The invasive mealybug *Phenacoccus solenopsis* Tinsley, a threat to tropical and subtropical agricultural and horticultural production systems—A review. Crop Prot..

[66] Muniappan R., Shepard B.M., Watson G.W., Carner G.R., Saritami D., Rauf A., Hammig M.D. (2008). First report of the papaya mealybug, *Paracoccus marginatus* (Hemiptera: Pseudococcidae), in Indonesia and India. J. Agric. Urban Entomol..

[67] Poorani J., Anuradha C., Thanigairaj R., Prashina M.P. (2024). Coccinellid predators of mealybugs infesting banana in South India, including a new species and a new record of *Scymnus* Kugelann (Coleoptera: Coccinellidae), with notes on other natural enemies. Zootaxa.

[68] Mani M., Joshi S., Kalyanasundaram M., Shivaraju C., Krishnamoorthy A., Asokan R., Rebijith K.B. (2013). A new invasive Jack Beardsley mealybug, *Pseudococcus jackbeardsleyi* (Hemiptera: Pseudococcidae) on papaya in India. Flor. Entomol..

[69] Mani M., Shylesha A.N. (2021). Invasive mealybugs and their management in India: Past, present and future. Hexapoda.

[70] Shylesha A.N., Joshi S. (2012). Occurrence of Madeira mealybug, *Phenacoccus madeirensis* Green (Hemiptera: Pseudococcidae) on cotton in India and record of associated parasitoids. J. Biol. Control.

[71] Nandudkar S.V., Santhakumar M.V., Chavan S.S., Jadhav A.D. (2025). Biology of *Nephus regularis* (Sicard) on the invasive mealybug, *Phenacoccus madeirensis* (Green) (Hemiptera: Pseudococcidae). Res. Perspect. Biol. Sci..

[72] Lepakshi N.M. (2015). Studies on the Bio-Ecology of Mealybug, *Phenacoccus madeirensis* Green (Homoptera: Pseudococcidae) with Special Reference to Cotton. Master’s Thesis.

[73] Soumya K.C., Sajeev T.V., Maneetha T.K., Vijayan K., Mathew G. (2013). Incidence of *Erionota thrax* (Hübner) (Lepidoptera: Hesperiidae) as a pest of banana in Kerala. Entomon.

[74] Poorani J., Padmanaban B., Sharanabasappa D., Thanigairaj R., Ragesh G. (2020). A review of the pest status and natural enemy complex of banana skipper *Erionota torus* Evans in South India and its management. Indian J. Entomol..

[75] Abdul-Jaleel K., Ghosh S.M., Jacob S., Anangh M.K. (2024). *Megaselia scalaris* (Loew) (Diptera: Phoridae)—A new pupal parasitoid of the invasive banana skipper *Erionota torus* Evans from India confirmed by COI gene barcoding. J. Adv. Zool..

[76] Sridhar V., Chakravarthy A.K., Asokan R., Vinesh L.S., Rebijith K.B., Vennila S. (2014). New record of the invasive tomato leaf miner, *Tuta absoluta* (Meyrick) (Lepidoptera: Gelechiidae) in India. Pest Manag. Hort. Ecosys..

[77] Ballal C., Gupta A., Mohan M., Lalitha Y., Verghese A. (2016). The new invasive pest *Tuta absoluta* (Meyrick) (Lepidoptera: Gelechiidae) in India and its natural enemies along with elevation of trichogrammatids for its biological control. Curr. Sci..

[78] Dubey A.K., Sundararaj R. (2015). A new combination and first record of the genus *Aleurothrixus* Quaintance and Baker (Hemiptera: Aleyrodidae) from India. Biosystmatica.

[79] Yadav G.A.K., Vasundhara J., Sumalatha B.V., Rameskumar A., Selvaraj K. (2025). Occurrence of exotic parasitoid *Encarsia cubensis* (Hymenoptera: Aphelinidae) on neotropical woolly whitefly *Aleurothrixus floccosus* and solanum whitefly *Aleurotrachelus trachoides* in India. Phytoparasitica.

[80] Tyagi K., Kumar V., Singha D., Chakraborty R. (2015). Morphological and DNA barcoding evidence for invasive pest thrips, *Thrips parvispinus* (Thripidae: Thysanoptera), newly recorded from India. J. Insect Sci..

[81] Thorat S.S., Sisodiya D.B., Gangwar R.K. (2022). Invasive thrips, *Thrips parvispinus* (Karny) an invasive threat: A review. Environ. Ecol..

[82] Sundararaj R., Selvaraj K. (2017). Invasion of rugose spiraling whitefly, *Aleurodicus rugiosperculatus* Martin (Hemiptera: Aleyrodidae) a potential threat to coconut in India. Phytoparasitica.

[83] Selvaraj K., Sundararaj R., Venkatesan T., Ballal C.R., Jalali S.K., Gupta A., Mrudula H.K. (2016). Potential natural enemies of the invasive rugose spiraling whitefly, *Aleurodicus rugioperculatus* Martin in India. J. Biol. Control.

[84] Poorani J., Thanigairaj R. (2017). First report of *Encarsia dispersa* Polaszek (Hymenoptera: Aphelinidae) as a parasitoid of rugose spiralling whitefly, *Aleurodicus rugioperculatus* Martin (Hemiptera: Aleyrodidae), a recent invasive pest in India, with notes on its predators. J. Biol. Control.

[85] Josephrajkumar A., Mohan C., Babu M., Krishna A., Krishnakumar V., Hegde V., Chowdappa P. (2019). First record of the invasive Bondar’s nesting whitefly, *Paraleyrodes bondari* Peracchi on coconut from India. Phytoparasitica.

[86] Sadhana V., Senguttuvan K., Murugan M., Suriya S. (2023). Review on exotic Bondar’s nesting whitefly, *Paraleyrodes bondari* Peracchi infestation on multiple cropping system. Phytoparasitica.

[87] Sadhana V., Senguttuvan K., Murugan M. (2022). Taxonomy of whiteflies’ natural enemies in Tamil Nadu cotton ecosystem. J. Curr. Crop Sci. Technol..

[88] Shylesha A.N., Jalali S.K., Gupta A., Varshney R., Venkatesan T., Shetty P., Ojha R., Ganiger P.C., Navik O., Subaharan K. (2018). Studies on new invasive pest *Spodoptera frugiperda* (J. E. Smith) (Lepidoptera: Noctuidae) and its natural enemies. J. Biol. Control.

[89] Hokkanen H., Pimentel D. (1984). New approach for selecting biological control agents. Can. Entomol..

[90] Muniappan R., Sah L.P., Nyaupane S., Bhandari G., Tiwari S., Bajracharya A.S.R., Gupta A., Subaharan K. (2024). Insect parasitoids of fall armyworm (Lepidoptera: Noctuidae) in Africa and Asia and their adoption in integrated pest management of maize in Nepal. J. Integr. Pest Manag..

[91] Selvaraj K., Sundararaj R., Sumalatha B.V. (2019). Invasion of the palm infesting whitefly, *Aleurotrachelus atratus* Hempel (Hemiptera: Aleyrodidae) in the Oriental region. Phytoparasitica.

[92] Selvaraj J., Rameshkumar A., Sumalatha B.V., Swathi H.D., Sardar S., Kazmi S.I. (2023). First report of *Encarsia cubensis* Gahan (Hymenoptera: Aphelinidae) an exotic parasitoid on the neotropical whitefly *Aleurotrachelus atratus* Hempel (Hemiptera: Aleyrodidae) in India. Phytoparasitica.

[93] Sundararaj R., Selvaraj K., Kalleshwaraswamy C.M., Ranjith M., Sumalatha B.V. (2020). First record of the invasive woolly whitefly *Aleurothrixus floccosus* (Maskell) from India. Indian J. Entomol..

[94] Selvaraj K., Sumalatha B.V. (2021). Distribution, biology and population dynamics of *Aleurothrixus floccosus* on guava. Indian J. Plant Prot..

[95] Joshi S., Pai S.G., Deepthy K.B., Ballal C.R., Watson G.W. (2020). The cassava mealybug, *Phenacoccus manihoti* Maltie-Ferrero (Hemiptera: Coccomorpha: Pseudococcidae) arrives in India. Zootaxa.

[96] Sampathkumar M., Mohan M., Shylesha A.N., Joshi S., Venkatesan T., Gupta A., Vennila S., Venkatachalam S.R., Vijayakumar M., Subramanian M. (2021). Occurrence of cassava mealybug, *Phenacoccus manihoti* Matile-Ferrero (Pseudococcidae: Hemiptera), a new invasive pest on cassava in India and prospects for its classical biological control. Curr. Sci..

[97] Sampathkumar M., Mohan M., Shylesha A.N., Joshi S., Gupta A., Saravanan P.A., Venkatachalam S.R. (2022). Mass Production and Release Technique of Anagyrus lopezi Wasp for Classical Biological Control of Invasive Cassava Mealybug in Cassava (tapioca) Plantations.

[98] Gupta A., Sampathkumar M., Mohan M., Shylesha A.N., Venkatesan T., Shashank P.R., Dhanyakumar O., Ramkumar P. (2021). Assessing adverse impact of the native biological control disruptors in the colonies of the recent invasive pest *Phenacoccus manihoti* Matile-Ferrero (Hemiptera: Pseudococcidae) in India. Glob. Ecol. Conserv..

[99] Mir S.H., Nugnes F., Bernardo U. (2024). First record of the invasive walnut leaf miner *Caloptilia roscipennella* (Lepidoptera: Gracillariidae) in Kashmir, India. J. Appl. Entomol..

[100] Selvaraj K., Yadav G.A.K., Vasundhara J., Sumalatha B.V., Sundararaj R., Sushil S.N. (2025). Record of the invasive Annona whitefly *Aleurotrachelus anonae* (Corbett) (Hemiptera: Aleyrodidae) from India. Curr. Sci..

[101] Muniappan R., Viraktamath C.A. (1986). Status of biological control of the weed, *Lantana camara* in India. Trop. Pest Manag..

[102] Sharma K.C., Chhetri G.K.K. (1977). Reports on studies on the biological control of *Eupatorium adenophorum*. Nepal. J. Agric..

[103] Wan T., Wang R. (1991). Achivements of biological weed control in the world and its prospects in China. Chin. J. Biol. Control.

[104] Swaminathan S., Raman A. (1981). On the morphology of the stem-galls of *Eupatorium adenophorum* Spreng. (Compositae) and the natural enemies of the cecidozoan, *Procecidochares utilis* Stone (Tephritidae, Diptera). Curr. Sci..

[105] Giriraj C.N., Bhat K.V. (1970). Supply of Natural Enemies of the “Siam Weed” Eupatorium odoratum (for Nigeria and Malaysia).

[106] Chacko M.J., Narasimham A.U. Biocontrol attempts against *Chromolaena odorata* in India—A review. Proceedings of the First International Workshop on Biological Control of Chromolaena odorata.

[107] Arjun C.P., Sarojkumar V., Sooraj V.R., Jaisankar R., Joseph A. (2016). Reoccurrence of *Pareuchaetes pseudoinsulata* Rego Barros (Arctiidae: Lepidoptera) population in Thiruvananthapuram district of Kerala, India: A biological control agent of the weed, *Chromolaena odorata* (Asteraceae). J. Biol. Control.

[108] Balaji R.K., Chitra N., Daniel A.J., Muthukumar M., Divya R. (2019). Occurrence of *Pareuchaetes pseudoinsulata* Rego Barros, 1956 (Arctiinae: Erebidae: Lepidoptera) in Pulney hills, Tamil Nadu. Ann. Plant Prot. Sci..

[109] Muniappan R., Bamba J., Spencer N.R. (2000). Biological control of *Chromolaena odorata*: Successes and failures. Proceedings of the X International Symposium on Biological Control of Weeds.

[110] Bhumannavar B.S., Ramani S., Rejeshwari S.K. (2007). Field release and impact of *Cecidochares connexa* (Macquart) (Diptera: Tephritidae) on *Chromolaena odorata* (L.) King and Robinson. J. Biol. Control.

[111] Sushilkumar (2015). History, progress and prospects of classical biological control in India. Indian J. Weed Sci..

[112] NBAIR *Pareuchaetes pseudoinsulata Rego Barros*; ICAR-National Bureau of Agricultural Insect Resources, Bengaluru, India, 2013; 1p. https://databases.nbair.res.in/Featured_insects/Pareuchaetes-pseudoinsulata.php.

[113] Jayanth K.P. (1987). Introduction and establishment of *Zygogramma bicolorata* on *Parthenium hysterophorus* at Bangalore, India. Curr. Sci..

[114] SreeramaKumar P. (2024). The exotic rust fungus *Puccinia abrupta* var. *partheniicola* on the invasive alien weed *Parthenium hysterophorus* in India: Rediscovery and first report of an epiphytotic. CABI Agric. Biosci..

[115] Shah N.C. (2018). *Eichhornia crassipes* (Pontederiaceae): An exotic aquatic menace in India and its possible uses. J. Econ. Taxon. Bot..

[116] Jayanth K.P., Visalakshy P.N.K. (1989). Establishment of the exotic mite *Orthogalumna terebrantis* Wallwork on Water hyacinth in Bangalore, India. J. Biol. Control.

[117] Waterhouse D.F., Norris K.R. (1987). Biological Control Pacific Prospects.

[118] Joy P.J. (1978). Ecology and Control of Salvinia (African Payal) the Molesting Weed of Kerala.

[119] Joy P.J., Varghese K.C., Abraham C.C. Studies on biology and host range of *Paulinia acuminata* de Geer (Orthoptera: Acrididae) and its efficiency for the control of *Salvinia molesta* Mitchell an aquatic floating weed in Kerala. Proceedings of the 8th Asia Pacific Weed Science Society Conference.

[120] ICAR (Indian Council of Agricultural Research) (2022). Biological Control of Alien Invasive Weed *Salvinia molesta* in Central India. https://www.icar.org.in/en/node/5865.

[121] ICAR (Indian Council of Agricultural Research) (2024). Biological Control of *Salvinia molesta* in Satpura Water Reservoir at Sarni, Madhya Pradesh. https://www.global-agriculture.com/india-region/biological-control-of-salvinia-molesta-in-satpura-water-reservoir-at-sarni-madhya-pradesh/.

[122] Pinjarkar V. 6 Weed-Infested Lakes in Chanda, G’Chiroli on Verge of Revival. Times of India/April 15, 2023. https://timesofindia.indiatimes.com/city/nagpur/6-weed-infested-lakes-in-chanda-gchiroli-on-verge-of-revival/articleshow/99506759.cms.

[123] Napompeth B. Biological control research and development in Thailand. Proceedings of the International Conference on Plant Protection in the Tropics.

[124] Waterhouse D.F. (1994). Biological Control of Weeds: Southeast Asian Prospects.

[125] Jayaraj N. (2025). Biocontrol Agents as a Solution for Invasive Species.

[126] Ellison C., Day M. (2010). Current status of release of *Puccinia spegazzinii* for *Mikania micrantha* control. Biocontrol News Info.

[127] Sankaran K.V., Ellison C.A., Suresh T.A., Zachariades C., Strathie L.W., Day M.D., Muniappan R. (2013). Biological control of *Mikania micrantha* in India: Opportunities and constraints. Proceedings of the Eighth International Workshop on Biology, Control and Manage. Chromolaena odorata and other Eupatorieae.

[128] Muniappan R., Viraktamath C.A. (1993). Invasive alien weeds in the Western Ghats. Curr. Sci..

[129] Priyadarshini S., Sahu S.A. (2025). New note on the distribution of *Mimosa diplotricha* C. Wright (Fabaceae) in Eastern India. Vegetos.

[130] Kuniata L.S., Muniappan R., Reddy G.V.P., Raman A. (2009). *Mimosa diplotricha* C. Wright ex Sauvalle (Mimosaceae). Biological Control of Tropical Weeds Using Arthropods.

[131] Kumar S. (2009). Biological control of parthenium in India: Status and prospects. Indian J. Weed Sci..

[132] Selvaraj K., Gupta A., Venkatesan T., Jalali S.K., Sundararaj R., Ballal C.R. (2017). First record of invasive rugose spiraling whitefly *Aleurodicus rugioperculatus* Martin (Hemiptera: Aleyrodidae) along with parasitoids in Karnataka. J. Biol. Control.

[133] Sampathkumar M., Mohan M., Joshi S., Shylesh A.N., Varshney R., Sushil S.N. (2024). Managing the Invasive Mango Soft Scale in India—*Cryptolaemus* Swings into Action. ICAR-NBAIR Video Documentary. https://www.youtube.com/watch?v=s9Gbhhr-UDo.

[134] Kumar S., Ray P., Kumar S., Misra J.S. (2018). Weed biological control research in India: Progress and prospects. Fifty Years of Weed Research in India.

[135] Beeson C.F.C., Chatterjee N.C. (1940). Possibilities of control of lantana (*Lantana aculeata* Linn.) by indigenous insect pests. Indian For. Rec..

[136] Jayanth K.P., Visalakshy P.N.G. (1992). Suppression of the lantana bug, *Teleonemea scrupulosa* by *Erythmelus teleonemiae* in Bangaluru, India. FAO Plant Prot. Bull..

[137] NBAIR (2013). Zygogramma bicolorata Pallister.

[138] Myrick S., Norton G.W., Selvaraj K.N., Natarajan K., Muniappan R. (2014). Economic impact of classical biological control of papaya mealybug in India. Crop Prot..

[139] Selvaraj K., Sumalatha B.V., Venkatesan T., Shylesha A.N., Kandan A., Chalapathi Rao N.B.V., Visalakshi M., Sushil S.N. (2024). Biological Control of Invasive Rugose Spiraling Whitefly Aleurodicus rugioperculatus on Coconut and Oil Palm.

[140] Krishnamoorthy A., Mani M., Visalakshy P.N.G., Sithanantham S., Ballal C.R., Jalali S.K., Bakthavatsalam N. (2013). Egg parsitoids in vegetable crops ecosystem: Research status and scope for utilization. Biological Control of Insect Pests Using Egg Parasitoids.

[141] Gurr G., Wratten S. (2000). Biological Control: Measures of Success.

[142] Reddy A.A., Reddy M., Mathur V. (2024). Pesticide use, regulation and policies in Indian agriculture. Sustainability.

[143] Shafia A. Integrated control of coconut hispid beetle *Brontispa longissimia* (Gestro) in the Maldives. Proceedings of the Report of the Expert Consultation on Coconut Beetle Outbreak in APPPC Member Countries, Bangkok, RAP Publication.

[144] Rethinam P., Singh S.P. Current Status of the Coconut Beetle Outbreaks in the Asia-Pacific Region. https://www.fao.org/4/ag117e/AG117E04.htm.

[145] Marambe B., Amarasinghe L., Silva K., Gamage G., Dissanayake S., Seneviratne A. (2014). Distribution, biology and management of *Mimosa pigra* in Sri Lanka. Research andmanagement of Mimosa pigra.

[146] Welgama A., Florentine S., Roberts J. (2022). A global review of the woody invasive alien species *Mimosa pigra* (giant sensitive plant): Its biology and management implications. Plants.

