# Supplementary material for: Role of Biological Control in Management of Invasive Exotic Arthropod Pests and Weeds in India
Source: Insects. 2026 Jan 1;17(1):61. doi: 10.3390/insects17010061 (PMC12841612; doi:10.3390/insects17010061)
Supplement: Supplementary file 1 [file insects-17-00061-s001.zip › insects-4014165-supplementary.pdf]

insects

MDPI

Supplementary File

Table S1. List of invasive insect pests recorded in the past 20 years.

Sl. No.	Common Name	Scientific Name and Taxonomic Position	Year of First Report
1	Eucalyptus gall wasp	*Leptocybe invasa* (Fisher and LaSalle) (Hymenoptera: Eulophidae)	2006
2	Cotton mealybug	*Phenacoccus solenopsis Tinsley* (Hemiptera: Pseudococcidae)	2006
3	Papaya mealybug	*Paracoccus marginatus* Williams & Granara de Willink (Hemiptera: Pseudococcidae)	2008
4	Madeira mealybug	*Phenacoccus madeirensis* Green (Pseudococcidae: Hemiptera)	2012
5	American Pinhole Borer (on rubber, Areca)	*Eulaptus parallelus* (Fabricius) (Coleoptera: Curculionidae: Platypodinae)	2012
6	Jack Beardsley mealybug	*Pseudococcus jackbeardsleyi* Gimpel and Miller (Pseudococcidae: Hemiptera)	2013
7	South American Tomato Pinworm	*Phthorimaea* (*Tuta*) *absoluta* (Meyrick) (Lepidoptera: Gelechiidae)	2014
8	Coconut Spindle infesting leaf beetle	*Wallacea* sp. (Chrysomelidae: Coleoptera)	2015
9	Western flower thrips	*Frankliniella occidentalis* (Pergande) (Thripidae: Thysanoptera)	2015
10	South East Asian Thrips	*Thrips parvispinus* (Karny) (Thysanoptera: Thripidae)	2015
11	Rugose spiralling whitefly	*Aleurodicus rugioperculatus* Martin (Hemiptera: Aleyrodidae)	2016
12	Legume whitefly	*Tetraleurodes acaciae* (Quaintance) (Hemiptera: Aleyrodidae)	2017
13	Solanum whitefly	*Aleurotrachelus trachoides* (Back) (Hemiptera: Aleyrodidae)	2018
14	Fall armyworm	*Spodoptera frugiperda* (J.E. Smith) (Lepidoptera: Noctuidae)	2018
15	Bondar's nesting whitefly	*Paraleyrodes bondari* Peracchi (Hemiptera: Aleyrodidae)	2018
16	Neotropical nesting whitefly	*Paraleyrodes minei* Iaccarino (Hemiptera: Aleyrodidae)	2018
17	Woolly whitefly	*Aleurothrixus floccosus* (Maskell) (Hemiptera: Aleyrodidae)	2019
18	Neotropical whitefly	*Aleurotrachelus atratus* Hempel (Hemiptera: Aleyrodidae)	2019
19	Cassava mealybug	*Phenacoccus manihoti* Matile-Ferrero (Hemiptera: Pseudococcidae)	2020
20	Apple leaf blotch miner	*Leucoptera malifoliella* (Costa) (Lyonetiidae: Lepidoptera)	2023
21	Mango soft scale	*Fistulococcus pokfulamensis* Hodgson & Martin (Hemiptera: Coccidae)	2023
22	Walnut leaf miner	*Caloptilia roscipennella* (Hübner) (Lepidoptera: Gracillariidae)	2024
23	Annona Whitefly	*Aleurotrachelus anonae* (Corbett) (Hemiptera: Aleyrodidae)	2024
24	Cactus mealybug	*Hypogeococcus pungens* Granara de Willink (Hemiptera: Pseudococcidae)	2024
25	Nesting Whitefly	*Paraleyrodes pseudonaranjae* Martin (Hemiptera: Aleyrodidae)	2025

https://doi.org/10.3390/insects17010061
